# LINC00511 enhances LUAD malignancy by upregulating GCNT3 via miR-195-5p

**DOI:** 10.1186/s12885-022-09459-7

**Published:** 2022-04-10

**Authors:** Youyi Zhang, Ping Xiao, Xiaobo Hu

**Affiliations:** 1 Department of Radiology, Sichuan Academy of Medical Sciences, Sichuan Provincial People’s Hospital, Chengdu, 610072 Sichuan China; 2 Department of Thoracic Surgery, Sichuan Cancer Hospital&Institute, Sichuan Cancer Center, School of Medicine, University of Electronic Science and Technology of China, Chengdu, 610041 Sichuan China; 3 Department of Respiratory Diseases, Chengdu First People’s Hospital, No. 18, North Wanxiang Road, Gaoxin District, Chengdu, 610016 Sichuan China

**Keywords:** LINC00511, miR-195-5p, GCNT3, LUAD

## Abstract

**Background:**

Accumulating evidence suggests that LINC00511 acts as an oncogenic long non-coding RNA (lncRNA) in various cancers, including lung adenocarcinoma (LUAD). Hence, we attempted to elucidate the potential role of LINC00511 in LUAD.

**Methods:**

LINC00511, miR-195-5p, and GCNT3 expression in LUAD was detected by qRT-PCR. Changes in the proliferation, migration, and invasion of LUAD cells after abnormal regulation of LINC00511, miR-195-5p, or GCNT3 were detected by CCK-8, BrdU, wound healing, and transwell assays. Bax and Bcl-2 protein expression was measured by western blotting. Additionally, we identified the targeting effects of LINC00511, miR-195-5p, and GCNT3 using luciferase and RNA immunoprecipitation (RIP) assays.

**Results:**

LINC00511 and GCNT3 were found to be upregulated in LUAD, while miR-195-5p was downregulated. Silencing LINC00511 or GCNT3 decreased the proliferation, migration, invasion, and Bcl-2 protein content in LUAD cells and increased the expression of Bax. Interference with miR-195-5p promoted malignant proliferation of cancer cells. miR-195-5p expression was affected by LINC00511and targeted GCNT3.

**Conclusion:**

Silencing LINC00511 promotes GCNT3 expression by inhibiting miR-195-5p and ultimately stimulates the malignant progression of LUAD.

**Supplementary Information:**

The online version contains supplementary material available at 10.1186/s12885-022-09459-7.

## Introduction

Lung adenocarcinoma (LUAD) is the most common subtype of lung cancer with the highest mortality, accounting for nearly 40% of all lung cancer cases [1, 2]. Despite remarkable achievements in the exploration and treatment of LUAD pathogenesis, morbidity and mortality have increased worldwide in recent years due to its high resistance to conventional radiotherapy and chemotherapy. Moreover, the overall survival rate of LUAD patients is less than 5 years [3, 4]. Therefore, understanding the molecular pathways driving LUAD progression and its associated targets is urgently needed.

Long non-coding RNAs (lncRNAs) are RNA molecules exceeding 200 nucleotides in length, and are emerging as crucial players in the progression of different diseases [5]. Abnormally expressed lncRNAs play carcinogenic or anticancer roles during the development of malignant tumors. It has been reported that lncRNAs mediate many tumor-related mechanisms such as transcriptional and translational regulation, protein modification, and RNA-protein or protein-protein complex formation [6]. The mRNA-miRNA-lncRNA-competing endogenous RNA network can be used as a biomarker for the prognosis of various human cancers [7]. For instance, the lncRNA HOTAIR enhances breast cancer cell growth and metastasis by negatively regulating miR-20a-5p and positively regulating HMGA2 [8]. LINC01133 inhibits the occurrence and metastasis of gastric cancer through the regulation of adenomatous polyposis coli (APC) gene expression and the Wnt-β-catenin pathway by competitive adsorption of miR-106a-3p, and is associated with good prognosis in gastric cancer patients [9]. A growing number of studies have shown that lncRNAs are significantly involved in LUAD. For example, lncRNA HOXA11-As, as a competitive endogenous RNA (ceRNA), promotes cisplatin resistance in LUAD cells through miR-454-3p/STAT3, and predicts a shorter survival time for LUAD patients with high expression of this lncRNA [10]. Moreover, LINC00511 has been shown to be overexpressed in LUAD and inhibits cell proliferation and tumorigenesis [11]. It has been suggested that understanding the functional mechanism of LINC00511 in LUAD may be helpful in developing new and effective molecular markers for this disease.

MicroRNAs (miRNAs) negatively regulate post-transcriptional gene expression, thereby modulating metastasis and carcinogenesis by altering the expression of tumor suppressors or oncogenes [12, 13]. miR-195-5p is a well-studied miRNA. In recent years, several studies have revealed that miR-195-5p plays a tumor-suppressive role in lung cancer by inhibiting the biological functions of glycolysis and proliferation of cells, thus indicating a good prognosis of lung cancer patients [14, 15]. Glucosaminyl (N-acetyl) transferase 3, mucin type (GCNT3), is a novel core mucin synthase [16] that plays a role as a cancer-promoting factor in gastric [16], colon [17], ovarian [18], and lung [19] cancers. However, there are few reports on the function of LINC00511/miR-195-5p/ GCNT3 axis in LUAD.

This study demonstrated that LINC00511 plays a crucial role in LUAD and analyzed the effects of different LINC00511/miR-195-5p/GCNT3 regulatory strategies on LUAD cell function. This widens the field of vision for molecular targets and therapeutic strategies for LUAD.

## Materials and Methods

### Human tissues and cell culture

A total of 40 pairs of LUAD tissues and adjacent cancer-free tissues were collected from LUAD patients treated in our hospital. The study was approved by the Ethics Committee of our hospital, and all the subjects signed the informed consent form. Two independent oncologists estimated the staging of the disease according to the 8th edition of the TNM classification for lung cancer [20]. Patients were classified as follows: 8 cases of stage I, 17 cases of stage II, 11 cases of stage III, and 3 cases of stage IV. All surgically resected samples were immediately transferred to liquid nitrogen and stored at −80 °C until further analysis.

A549, Calu-3 (human LUAD cells), and BEAS-2B (human bronchial epithelial cells) were purchased from ATCC (USA) and cultured in DMEM (HyClone, USA). The human LUAD cell lines DV-90 and PC-9 were purchased from Cobioer (China) and cultured in RPMI-1640 medium (HyClone). All media were supplemented with 10% FBS (HyClone), 100 mg/mL streptomycin (Gibco, USA) and 100 U/mL penicillin (Gibco). All cell lines were incubated at 37 °C with 5% CO_2_.

### qRT-PCR assay

Total miRNA was extracted using the mirVanaTM miRNA isolation kit (Ambion, USA). cDNA was synthesized from total RNA using miRNA-specific primers and the TaqMan miRNA reverse transcription kit (ABI, USA) according to the manufacturer’s instructions. Sequence-specific primers were used for PCR amplification performed on the Applied Biosystems 7500 real-time PCR system (Applied Biosystems, USA). U6 was used as a control to normalize miR-195-5p expression. Data were evaluated using the 2^-ΔΔCt^ method.

Total RNA was extracted using the RNA high-purity total RNA rapid extraction kit (Qiagen, Japan). The isolated total RNA was reverse transcribed using the PrimeScript RT reagent kit (TaKaRa, Japan). The mRNA expression levels of LINC00511 and GCNT3 were detected using the SYBR Premix Ex Taq II kit (TaKaRa). GAPDH was used as the internal reference control. The primers used are listed in Table 1.

**Table 1:** The PCR primers used in the presence work

Gene	Primer sequence (5′-3′)
LINC00511	Forward: CTAACAAGAGGGTAAGTGTCAG
	Reverse: AAGTCGACAACCCCATCGTTAC
miR-195-5p	Forward: GGGGTAGCAGCACAGAAAT
	Reverse: TCCAGTGCGTGTCGTGGA
GCNT3	Forward: GCCAGTAAGCTGGTTCGG
	Reverse: GCCAGTAAGCTGGTTCGG
GAPDH	Forward: GACAGTCAGCCGCATCTTCT
	Reverse: TTAAAAGCAGCCCTGGTGAC
U6	Forward: CACCAAGAAGTCTTCTCCTTCAGTG
	Reverse: GCTGAGAGGGTCCACAGCT

### Cell transfection

Specific siRNAs targeting LINC00511 (si-LNC), and GCNT3 (si-GCNT3), and control siRNA plasmid (si-NC) were synthesized by RiboBio (Guangzhou, China). The miR-195-5p mimic, miR-195-5p inhibitor, and the corresponding controls (mimic-NC, inhibitor-NC) were purchased from Switchgear Genomics (USA). PC9 and A549 cells were transfected with 50 nM si-LNC, 50 nM si-GCNT3, 100 nM miR-195-5p mimic, and 75 nM miR-195-5p inhibitor using Lipofectamine 2000 (Invitrogen, USA) according to the manufacturer’s instructions. After 48 h of transfection, qRT-PCR was performed to detect the transfection efficiency.

### Western blotting

Total proteins were isolated using RIPA lysis buffer (Thermo Fisher Scientific, Waltham, MA, USA) and quantified using the BCA protein analysis kit (Pierce, USA). Proteins were separated on a 10% gel by SDS-PAGE, and subsequently transferred onto PVDF membranes. The membranes were blocked with 5% skim milk at 25 °C for 1 h, and then incubated with primary antibodies, Bax (ab32503; Abcam, UK), Bcl-2 (ab32124; Abcam), GCNT3 (ab77728; Abcam), and GAPDH (ab8245; Abcam) overnight at 4 °C. After washing with PBST buffer (containing 0.1% Tween-20), the membranes were incubated with secondary antibodies (ab6721; Abcam) for 1 h at 25 °C. ECL substrates (Millipore, USA) were used for target protein detection, and ImageJ software was used for standard protein analysis.

### CCK-8 assay

PC9 and A549 cells were seeded into 96-well plates at a density of 1 × 10^4^ cells/well. After incubation for 0, 24, 48, or 72 h, CCK-8 solution (10 μL) (Dojindo Molecular Technologies, Japan) was added into each well. After 2 h of further incubation, a microplate reader (BioTek Instruments, USA) was used to measure the optical density (OD450) at each indicated time point.

### BrdU assay

DNA synthesis was measured by BrdU incorporation into PC9 and A549 cells to determine cell proliferation. PC9 and A549 cells (2 × 10^3^ cells/well) were seeded in a 96-well culture plate. After 48 h of culture, 10 μM BrdU (BD Pharmingen, USA) was added and the cells were incubated for another 2 h. The medium was removed and co-cultured with BrdU antibody (Sigma-Aldrich) for 60 min, followed by incubation with tetramethylbenzidine (peroxidase substrate) for 30 min. The absorbance of each well was determined at 450 nm.

### Wound healing assay

PC9 and A549 cells were collected and seeded in 6-well plates after transfection for 48 h. When the cells reached 90% confluency, the monolayer cell surface was scratched with a sterile 200 μL pipette tip. After washing with PBS, the cells were cultured in serum-free medium. Wound images were observed at the specified time points (0 h and 24 h). The scratch width (W) was determined, and the cell migration rate was calculated as (W0 h-W24 h)/W0 h × 100% [21].

### Transwell assay

Transwell chambers (Corning, NY, USA) pre-coated with 20 μg of Matrigel (Corning, NY, USA) were used to perform this assay. The cells were suspended in serum-free medium and then placed in the upper chamber, and the medium containing 10% FBS was placed in the lower chamber. The cells in the upper chamber were gently wiped off after incubation for 48 h. Cells in the lower chamber were fixed with methanol and stained with 0.1% crystal violet at 25 °C. Images were captured by a microscope (Olympus, Japan) to count the number of cells.

### Luciferase assay

Luciferase reporter plasmids, including wild-type and mutant pGLO-LINC00511 (LINC00511-WT and LINC00511-MUT), and wild-type and mutant pGLO-GCNT3 3′UTR (GCNT3-WT and GCNT3-MUT) were constructed by Sangon (China). The reporter plasmids were co-transfected with mimic-NC or miR-195-5p mimic into PC9 and A549 cells using Lipofectamine 2000. After 48 h of transfection, luciferase activity was detected using the dual-luciferase reporter kit (Promega, USA).

### RNA Immunoprecipitation (RIP) assay

The RIP RNA-binding protein immunoprecipitation kit (Millipore) was used for this analysis. Briefly, PC9 and A549 cells transfected with mimic-NC or miR-195-5p mimic were lysed and incubated with RIP buffer containing magnetic beads bound to Ago2 antibody or IgG (Millipore). LINC00511 expression was detected by qRT-PCR.

### Statistical analysis

The data were expressed as the mean ± SD and analyzed using GraphPad Prism (GraphPad, USA). Statistical differences between multiple groups were analyzed using one-way ANOVA. Student’s t-test was used for paired comparison. Statistical significance was set at P < 0.05.

## Results

### LINC00511/miR-195-5p/GCNT3 axis is a potential regulator of lung cancer via a ceRNA network

**Fig. 1:**
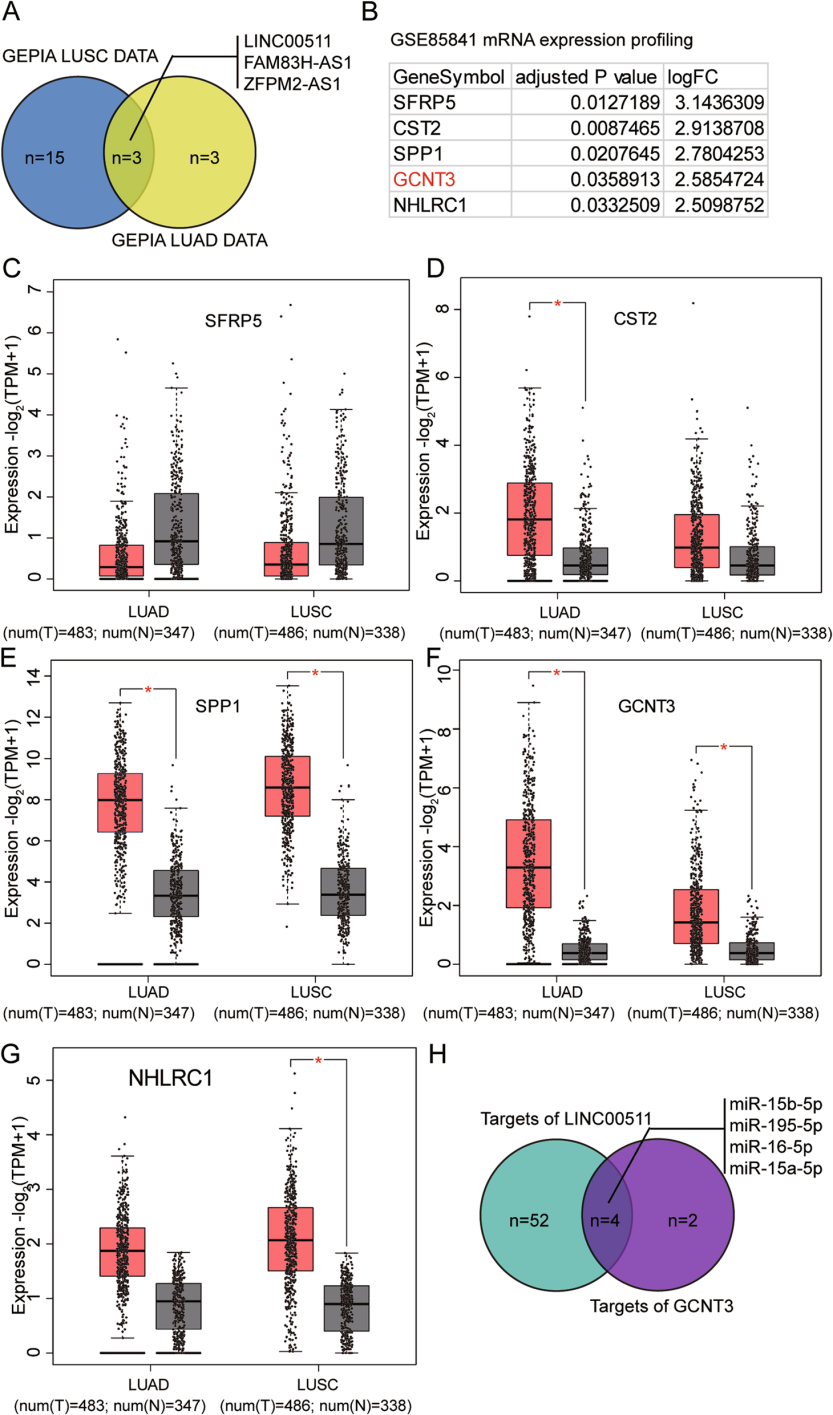
LINC00511/miR-195-5p/GCNT3 axis was identified to be a potential regulator in lung cancer via a ceRNA network. **A**. Common upregulated lncRNAs in LUAD and LUSC compared with normal non-tumor samples were screened by GEPIA 2 with adj. *P* < 0.05 and logFC>1.5. LUAD: lung adenocarcinoma. LUSC: lung squamous cell carcinoma. **B**. The top 5 most significantly upregulated genes from GSE85841 were presented. GSE85841 is a mRNA microarray data of 8 LUAD and adjacent non-tumor samples to screen the upregulated genes with *P* < 0.05 and logFC> = 1.5. **C**-**G**. The expression of the 5 genes in LUAD and LUSC. Data obtained from GEPIA database. **H**. The candidate miRNAs that link LINC00511 and GCNT3 mRNA were overlapped by Venny 2.1.0. The targets of LINC00511 and GCNT3 mRNA were both predicted using starbase algorithm

By examining the differentially expressed lncRNAs in both LUAD and lung squamous cell carcinoma (LUSC) samples compared with normal non-tumor samples with adjusted P < 0.05, and logFC>1.5, we identified three candidates by Gene Expression Profiling Interactive Analysis (GEPIA2) 2. In descending order of expression, these were LINC00511, FAM83H-AS1, and ZFPM2-AS1 (Fig. 1A). LINC00511 was the most significantly upregulated lncRNA and has been reported to be a significant driver of lung cancer [22–26]. In addition, we analyzed the gene expression profile of GSE85841 that included eight LUAD samples and adjacent non-tumor samples with adjusted P < 0.05 and logFC≥1.5, and researched the top five most significantly upregulated genes: SFRP5, CST2, SPP1, GCNT3, and NHLRC1 (Fig. 1B). According to the GEPIA database, SFRP5 expression did not differ between LUAD, LUSC, and non-tumor tissues (Fig. 1C). CST2 expression differed only between LUAD and non-tumor tissues (Fig. 1D), and NHLRC1 expression differed between LUSC and non-tumor tissues (Fig. 1G). SPP1 and GCNT3 were differentially expressed between LUAD or LUSC and non-tumor controls (Fig. 1E, F). While SPP1 has been studied extensively in lung cancer [27–30], GCNT3 has not been well-studied. Knockdown of GCNT3 has been reported to suppress lung cancer cell proliferation and mobility [19]. To seek the miRNA connecting LINC00511 and GCNT3, StarBase v.2.0 was used to predict the miRNAs binding to LINC00511 and GCNT3. Using Venny 2.1.0, it was found that four miRNAs were associated with LINC00511 and GCNT3: miR-15a-5p, miR-15b-5p, miR-16-5p, and miR-195-5p (Fig. 1H). miR-195-5p has been reported to be a tumor suppressor in lung cancer [14, 31–34]. Therefore, we were interested in examining whether miR-195-5p links LINC00511 and GCNT3, thus exerting cancer-suppressing functions in lung cancer.

### LINC00511 is upregulated in LUAD

**Fig. 2:**
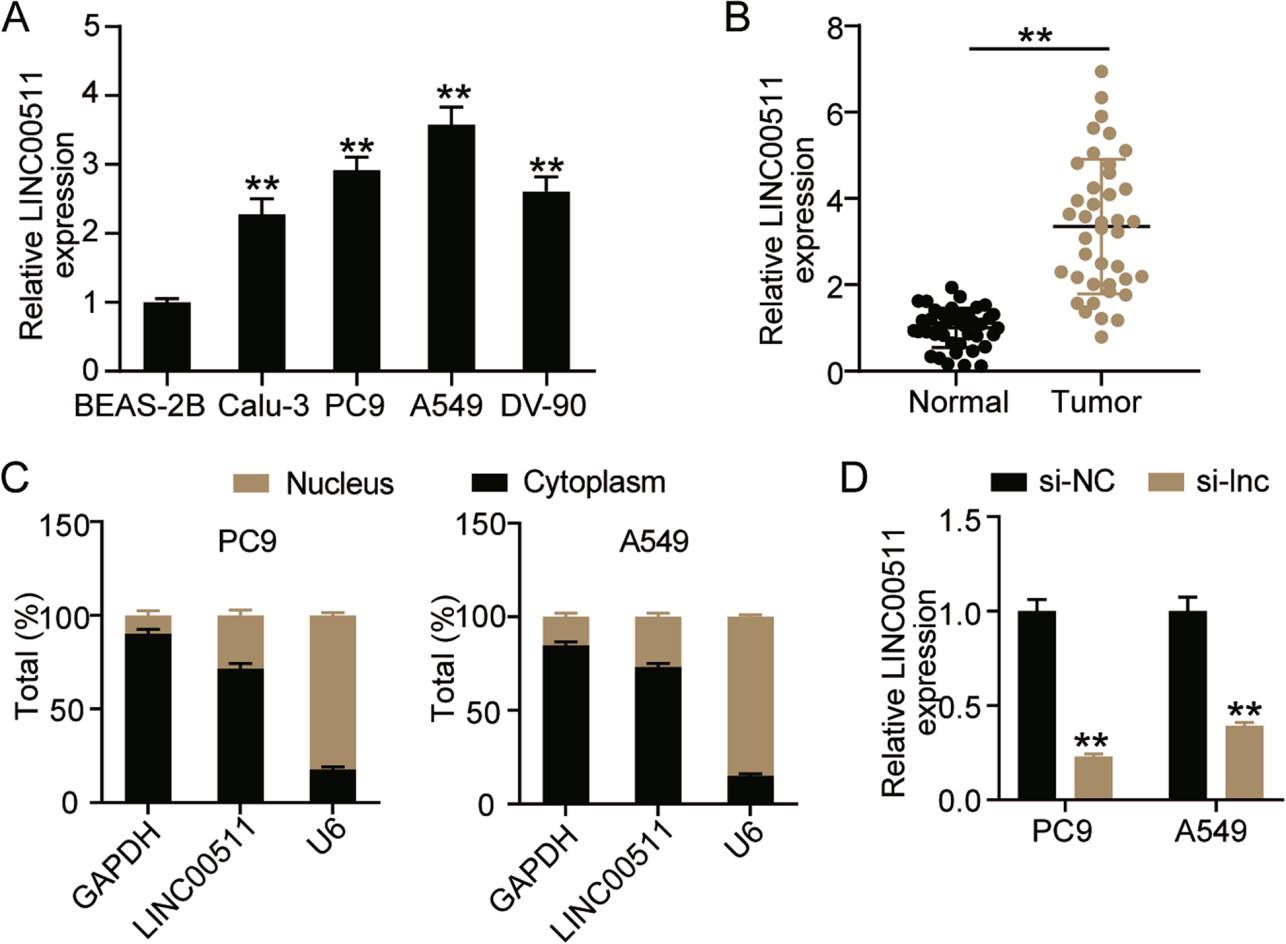
LINC00511 was upregulated in LUAD. **A** The expression of LINC00511 was detected by qRT-PCR assay in Human LUAD cell line (A549, Calu-3, DV-90 and PC-9) and human bronchial epithelial cell line BEAS-2B. **P < 0.001 compared with BEAS-2B. **B** The expression of LINC00511 was detected by qRT-PCR assay in cancer tissues (*n* = 40) and normal tissues (*n* = 40). **C** The subcellular localization assay for LINC00511. **D** The expression of LINC00511 was tested by qRT-PCR in A549 and PC9 cells transfected with si-NC and si-lnc for 48 h. **P < 0.001 compared with si-NC. si-lnc, siRNA of LINC00511. si-NC, negative control of si-RNA

LINC00511 has been identified as a lncRNA overexpressed in various cancers. Therefore, we explored the changes in LINC00511 expression in LUAD cells. At the cellular level, LINC00511 expression was found to be elevated in A549, Calu-3, DV-90, and PC9 cell lines compared with BEAS-2B cells, and the expression levels of LINC00511 were higher in A549 and PC9 cells (Fig. 2A). At the clinical level, the results revealed an approximately 3-fold increase in LINC00511 expression in LUAD tissues compared to normal tissues (Fig. 2B). In addition, the subcellular localization of LINC00511 was investigated. The data revealed that LINC00511 was abundant in the cytoplasm, indicating that it mainly functions in the cytoplasm (Fig. 2C). Furthermore, transfecting A549 and PC9 cells with LINC00511 specific siRNA showed a 60% decrease in LINC00511 expression levels in the si-LNC group compared to that in the si-NC group (Fig. 2D).

### Interference with LINC00511 inhibited the malignant proliferation of LUAD cells

**Fig. 3:**
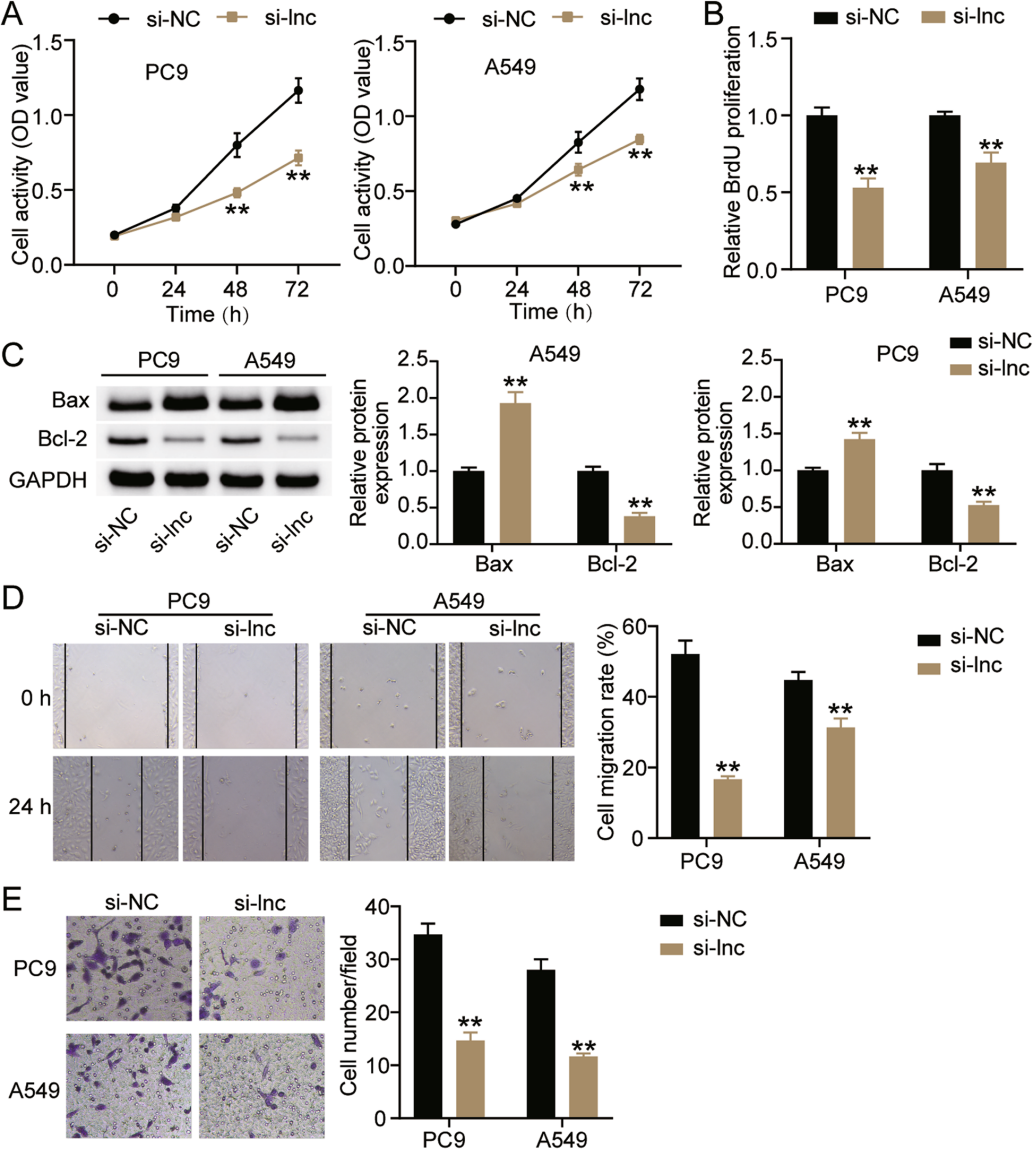
Interference with LINC00511 inhibited the malignant proliferation of LUAD ells. **A** Cell viability was detected by CCK-8 assay in A549 and PC9 cells transfected with si-NC and si-lnc for 0, 24, 48 or 72 h. **B** Cell proliferation was determined by BrdU assay in A549 and PC9 cells transfected with si-NC and si-lnc for 48 h. **C** Bax and Bl-2 protein expression detected by western blot assay in A549 and PC9 cells transfected with si-NC and si-lnc for 48 h. **D** Migration capacity was determined by wound healing assay in A549 and PC9 cells transfected with si-NC and si-lnc for 48 h. (E) Cell invasion ability was detected by transwell invasion assay in A549 and PC9 cells transfected with si-NC and si-lnc for 48 h. **P < 0.001 compared with si-NC. si-lnc, siRNA of LINC00511. si-NC, negative control of si-RNA

Next, we investigated the effect of LINC00511 on the biological functions of LUAD cells. The CCK-8 assay indicated that the viability of A549 and PC9 cells decreased to approximately 40% compared to that of the si-NC group after knockdown of LINC00511 (Fig. 3A). The BrdU assay showed that in comparison with the si-NC group, cell proliferation in the si-LNC group decreased by more than 30% (Fig. 3B). Western blotting revealed that interference with LINC00511 increased Bax protein levels in A549 and PC9 cells by 1.9-fold and 1.5-fold, respectively, and reduced Bcl-2 protein levels by 40 and 35%, respectively (Fig. 3C). In addition, the effects of LINC00511 on the metastasis of A549 and PC9 cells were monitored. The wound healing assay revealed that silencing LINC00511 reduced cell migration rates by more than 30% (Fig. 3D). Moreover, the transwell assay showed a 60% reduction in cell invasion in the si-LNC group compared to the si-NC group (Fig. 3E). These results suggested that low LINC00511 expression inhibited the malignant proliferation of LUAD cells.

### LINC00511 targeted and negatively regulated miR-195-5p

**Fig. 4:**
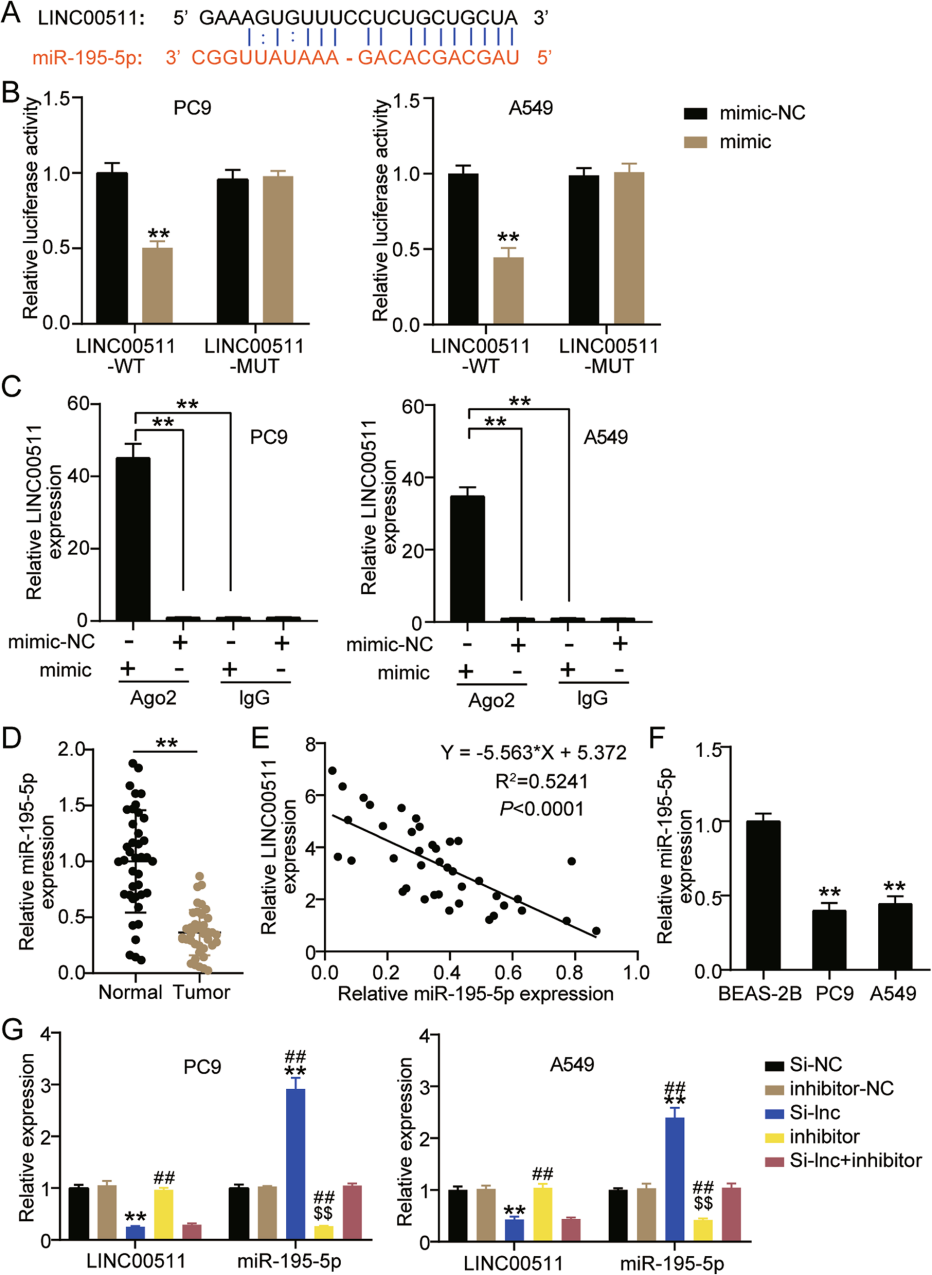
LINC00511 targeted and negatively regulated miR-195-5p. **A** The binding site of LINC00511 with miR-195-5p was confirmed according to bioinformatics analysis. **B** Luciferase reporter assay confirmed the molecular binding. **P < 0.001 compared with mimic-NC. **C** RIP was conducted to measure the enrichment of LINC00511 in Ago2 immunoprecipitate and IgG-pellet. **P < 0.001. **D** The expression of miR-195-5p was detected by qRT-PCR assay in cancer tissues (*n* = 40) and normal tissues (*n* = 40). **P < 0.001. **E** pearson analysis was used to analyze the expression of LINC00511 and miR-195-5p in cancer tissues. **F** The expression of miR-195-5p was detected by qRT-PCR assay in A549, PC9 and BEAS-2B cells. **P < 0.001 compared with BEAS-2B. **G** The LINC00511 and miR-195-5p expression were detected by qRT-PCR assay in A549 and PC9 cells transfected with si-lnc or inhibitor for 48 h. **P < 0.001 compared with si-NC group. $$P < 0.001 compared with inhibitor-NC. ##*P* < 0.001 compared with si-lnc + inhibitor. si-lnc, siRNA of LINC00511. si-NC, negative control of siRNA. inhibitor, miR-195-5p inhibitor. inhibitor-NC, negative control of inhibitor. si-lnc + inhibitor, siRNA of LINC00511 + miR-195-5p inhibitor

Next, the miRNA regulated by LINC00511 was identified. The Starbase website showed that LINC00511 contained a binding site for miR-195-5p (Fig. 4A). In addition, wild-type or mutant LINC00511 and miR-195-5p mimic were transfected into A549 and PC9 cells. It was found that the luciferase activity of cells transfected with LINC00511-WT and miR-195-5p mimic decreased by more than 50%, and the luciferase activity of cells transfected with LINC00511-MUT and miR-195-5p mimic did not change significantly (Fig. 4B). Additionally, the RIP assay showed that in A549 and PC9 cells transfected with miR-195-5p mimic, the expression of LINC00511 increased approximately 15 times after Ago2 treatment, compared to IgG (Fig. 4C). Next, in clinical samples, it was found that miR-195-5p expression in cancer tissues decreased by 70% (Fig. 4D). Pearson analysis revealed that LINC00511 was negatively correlated with miR-195-5p expression in cancer tissues (Fig. 4E). Further detection by qRT-PCR showed that miR-195-5p levels in A549 and PC9 cells were lower than that in BEAS-2B cells (Fig. 4F). Additionally, it was found that knockdown of LINC00511 expression upregulated miR-195-5p levels in A549 and PC9 cells, and silencing miR-195-5p downregulated miR-195-5p levels, and reversed the effect of LINC00511 knockdown on miR-195-5p levels (Fig. 4G).

### LINC00511 negatively regulated miR-195-5p to affect the malignant proliferation of LUAD cells

**Fig. 5:**
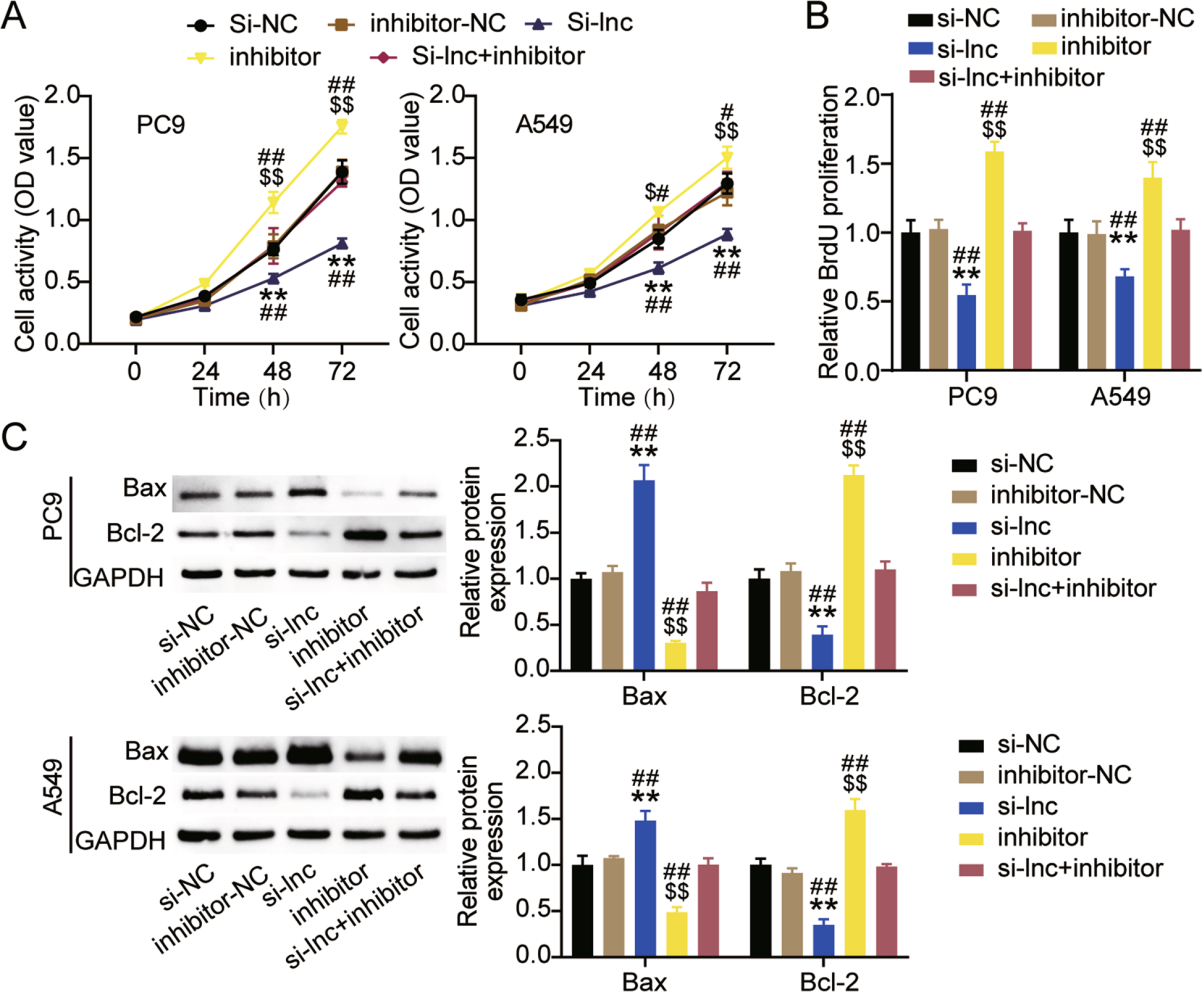
LINC00511 negatively regulated miR-195-5p to affect the proliferation and apoptosis of LUAD cells. **A** Cell viability was detected by CCK-8 assay in A549 and PC9 cells transfected with si-lnc or inhibitor for 0, 24, 48 or 72 h. **B** Cell proliferation was determined by BrdU assay in A549 and PC9 cells transfected with si-lnc or inhibitor for 48 h. **C**. Bax and Bl-2 protein expression detected by western blot assay in A549 and PC9 cells transfected with si-lnc or inhibitor for 48 h. **P < 0.001 compared with si-NC group. $*P* < 0.05, $$*P* < 0.001 compared with inhibitor-NC. #*P* < 0.05, ##*P* < 0.001 compared with si-lnc + inhibitor. si-lnc, siRNA of LINC00511. si-NC, negative control of siRNA. inhibitor, miR-195-5p inhibitor. inhibitor-NC, negative control of inhibitor. si-lnc + inhibitor, siRNA of LINC00511 + miR-195-5p inhibitor

**Fig. 6:**
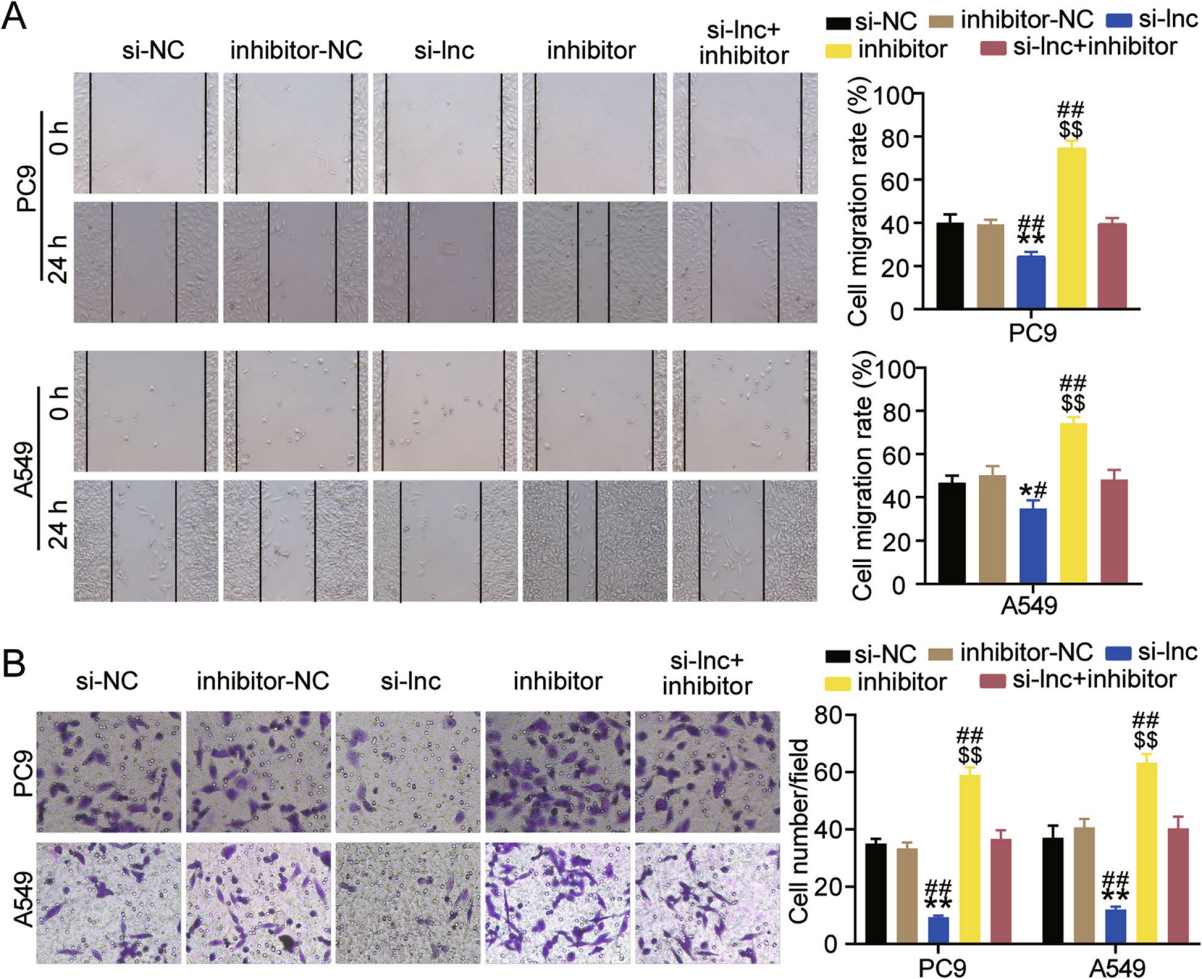
LINC00511 negatively regulated miR-195-5p to affect the metastasis of LUAD cells. **A** Migration capacity was determined by wound healing assay in A549 and PC9 cells transfected with si-lnc or inhibitor for 48 h. **B** Cell invasion ability was detected by transwell invasion assay in A549 and PC9 cells transfected with si-lnc or inhibitor for 48 h. *P < 0.05, **P < 0.001 compared with si-NC group. $$*P* < 0.001 compared with inhibitor-NC. #*P* < 0.05, ##*P* < 0.001 compared with si-lnc + inhibitor. si-lnc, siRNA of LINC00511. si-NC, negative control of siRNA. inhibitor, miR-195-5p inhibitor. inhibitor-NC, negative control of inhibitor. si-lnc + inhibitor, siRNA of LINC00511 + miR-195-5p inhibitor

Next, the functional changes in A549 and PC9 cells induced by LINC00511 or miR-195-5p were studied. As shown in Fig. 5A, low expression of miR-195-5p facilitated the survival of A549 and PC9 cells by approximately 1.3 times. When both LINC00511 and miR-195-5p were expressed at low levels, cell viability was restored (Fig. 5A). In addition, the BrdU assay revealed that interference with miR-195-5p augmented the proliferation of LUAD cells and eliminated the inhibitory effect of silencing LINC00511 (Fig. 5B). Western blot analysis showed that Bax protein levels were more than 50% lower in the inhibitor group than in the inhibitor-NC or si-LNC + inhibitor groups, and Bcl-2 protein levels were increased by more than 1.5 times (Fig. 5C). Moreover, both A549 and PC9 cells exhibited higher migration rates after miR-195-5p knockdown compared to the inhibitor-NC group or si-LNC + inhibitor group (Fig. 6A). The transwell assay indicated that low expression of miR-195-5p facilitated cell invasion by approximately 1.5-fold, thereby preventing the effect of low expression of LINC00511 on cell invasion (Fig. 6B). These results revealed that silencing miR-195-5p reversed the functional effects of LINC00511 in A549 and PC9 cells.

### GCNT3 is the target gene of miR-195-5p

**Fig. 7:**
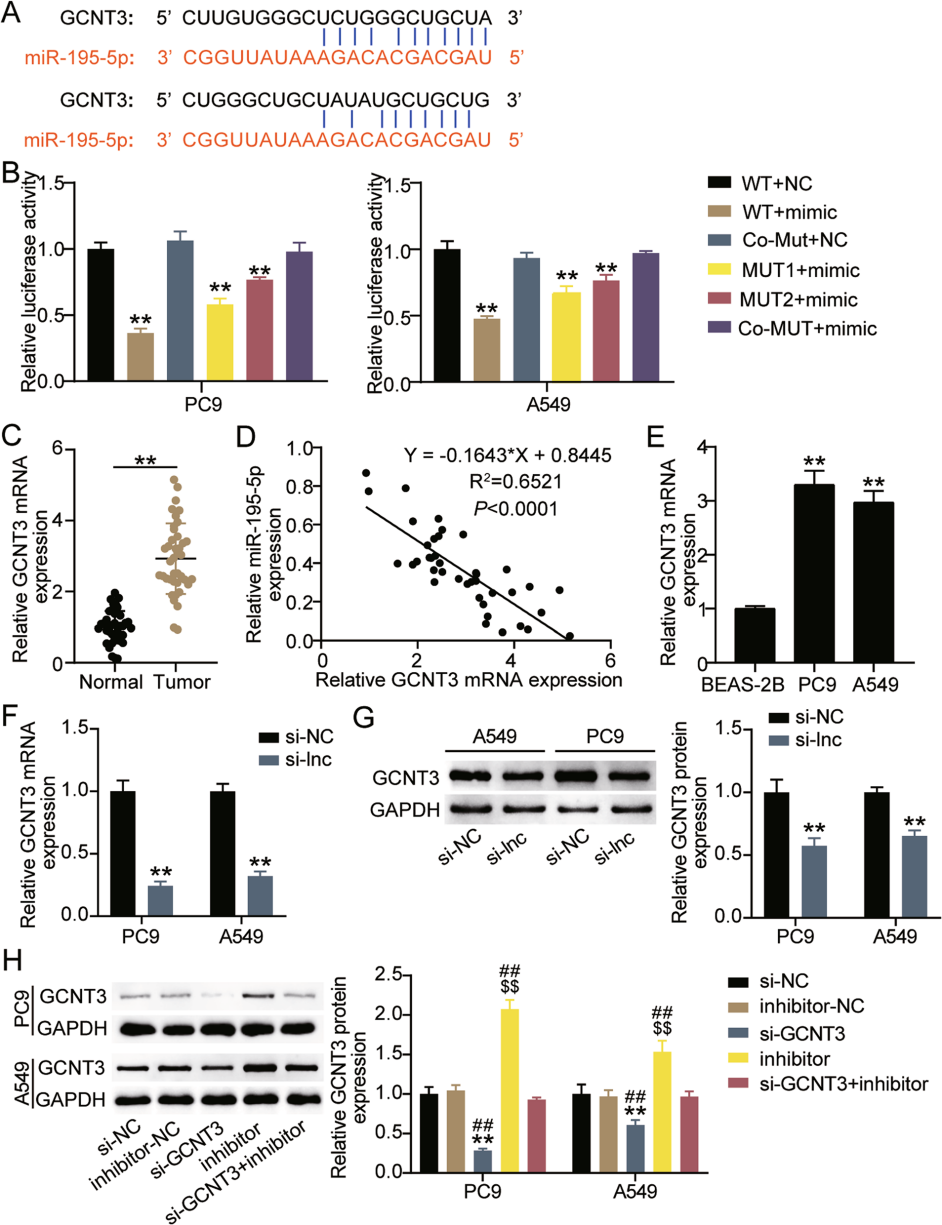
GCNT3 was the target gene of miR-195-5p. **A** The schematic diagram presents the complementary binding sites within GCNT3 and miR-195-5p. **B** Luciferase reporter assay confirmed the molecular binding. **P < 0.001 compared with WT + NC. #*P* < 0.05 compared with WT + mimic. **C** The expression of GCNT3 was detected by qRT-PCR assay in cancer tissues and normal tissues. **P < 0.001. **D** Pearson analysis was used to analyze the expression of GCNT3 and miR-195-5p in cancer tissues. **E** The expression of GCNT3 was detected by qRT-PCR assay in A549, PC9 and BEAS-2B cells. **P < 0.001 compared with BEAS-2B. **F** The GCNT3 mRNA expression were detected by qRT-PCR assay in A549 and PC9 cells transfected with si-lnc for 48 h. **G** The GCNT3 protein expression were detected by western blot assay in A549 and PC9 cells transfected with si-lnc for 48 h. **H** The GCNT3 protein expression were detected by western blot assay in A549 and PC9 cells transfected with si-GCNT3 or inhibitor for 48 h. **P < 0.001 compared with si-NC group. $$*P* < 0.001 compared with inhibitor-NC. ##*P* < 0.001 compared with si-GCNT3 + inhibitor. si-GCNT3, siRNA of GCNT3. si-NC, negative control of siRNA. inhibitor, miR-195-5p inhibitor. inhibitor-NC, negative control of inhibitor. si-GCNT3 + inhibitor, siRNA of GCNT3 + miR-195-5p inhibitor

The StarBase website showed that GCNT3 contained two binding sites for miR-195-5p (Fig. 7A). The luciferase activity of A549 and PC9 cells co-transfected with GCNT3-WT and miR-195-5p mimic decreased by more than 50%, and that of cells co-transfected with MUT1 and miR-195-5p mimic decreased by approximately 40%, and cells co-transfected with MUT2 and miR-195-5p mimic showed a 25% decrease in luciferase activity (Fig. 7B). It was observed that miR-195-5p binds to the 3′-UTR of GCNT3. qRT-PCR showed that GCNT3 levels were nearly 3 times higher in cancer tissues than in normal tissues (Fig. 7C). Additionally, there was an inverse correlation between GCNT3 and miR-195-5p in cancer (Fig. 7D). GCNT3 levels in A549 and PC9 cells were approximately 3 times higher than in BEAS-2B cells (Fig. 7E). Moreover, we investigated the regulatory effect of LINC00511 on GCNT3. qRT-PCR and western blot showed that the mRNA and protein levels of GCNT3 decreased by about 70 and 50% respectively in the si-lnc group compared with the si-NC group (Fig. 7F and G). Indicating that LINC00511 positively regulate the expression of GCNT3. In addition, GCNT3-specific siRNA and miR-195-5p inhibitor were transfected into A549 and PC9 cells. The GCNT3 protein expression increased after miR-195-5p downregulation, which was reduced after GCNT3 downregulation, which eliminated the impact of miR-195-5p downregulation on GCNT3 protein expression (Fig. 7H).

### GCNT3 silencing reversed the effect of miR-195-5p inhibitor on the malignant proliferation of LUAD cells

**Fig. 8:**
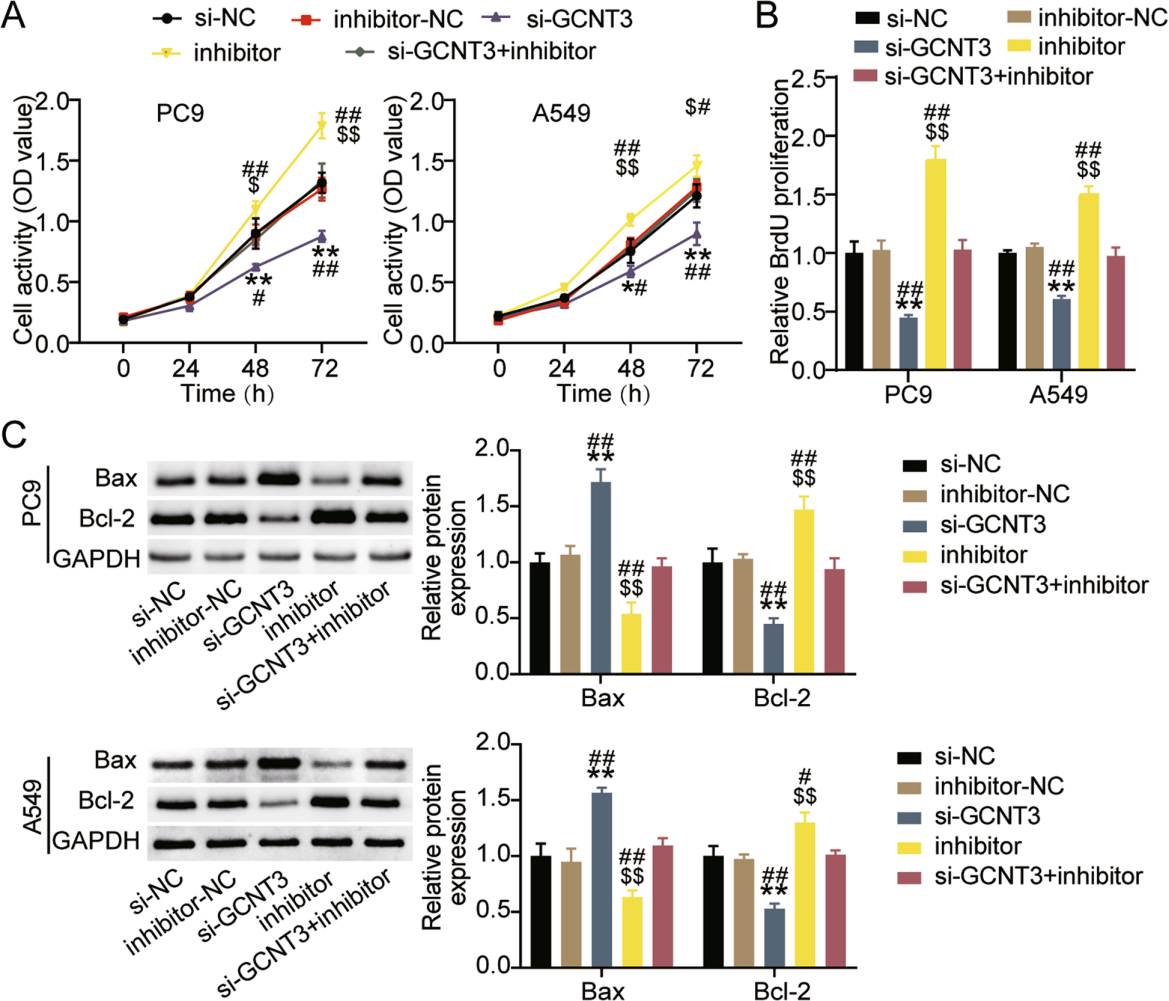
GCNT3 silencing reversed the effect of miR-195-5p on the proliferation and apoptosis of LUAD cells. **A** Cell viability was detected by CCK-8 assay in A549 and PC9 cells transfected with si-GCNT3 or inhibitor for 0, 24, 48 or 72 h. **B** Cell proliferation was determined by BrdU assay in A549 and PC9 cells transfected with si-GCNT3 or inhibitor for 48 h. **C** Bax and Bl-2 protein expression detected by western blot assay in A549 and PC9 cells transfected with si-GCNT3 or inhibitor for 48 h. *P < 0.05, **P < 0.001 compared with si-NC group. $*P* < 0.05, $$*P* < 0.001 compared with inhibitor-NC. #*P* < 0.05, ##*P* < 0.001 compared with si-GCNT3 + inhibitor. si-GCNT3, siRNA of GCNT3. si-NC, negative control of siRNA. inhibitor, miR-195-5p inhibitor. inhibitor-NC, negative control of inhibitor. si-GCNT3 + inhibitor, siRNA of GCNT3 + miR-195-5p inhibitor

**Fig. 9:**
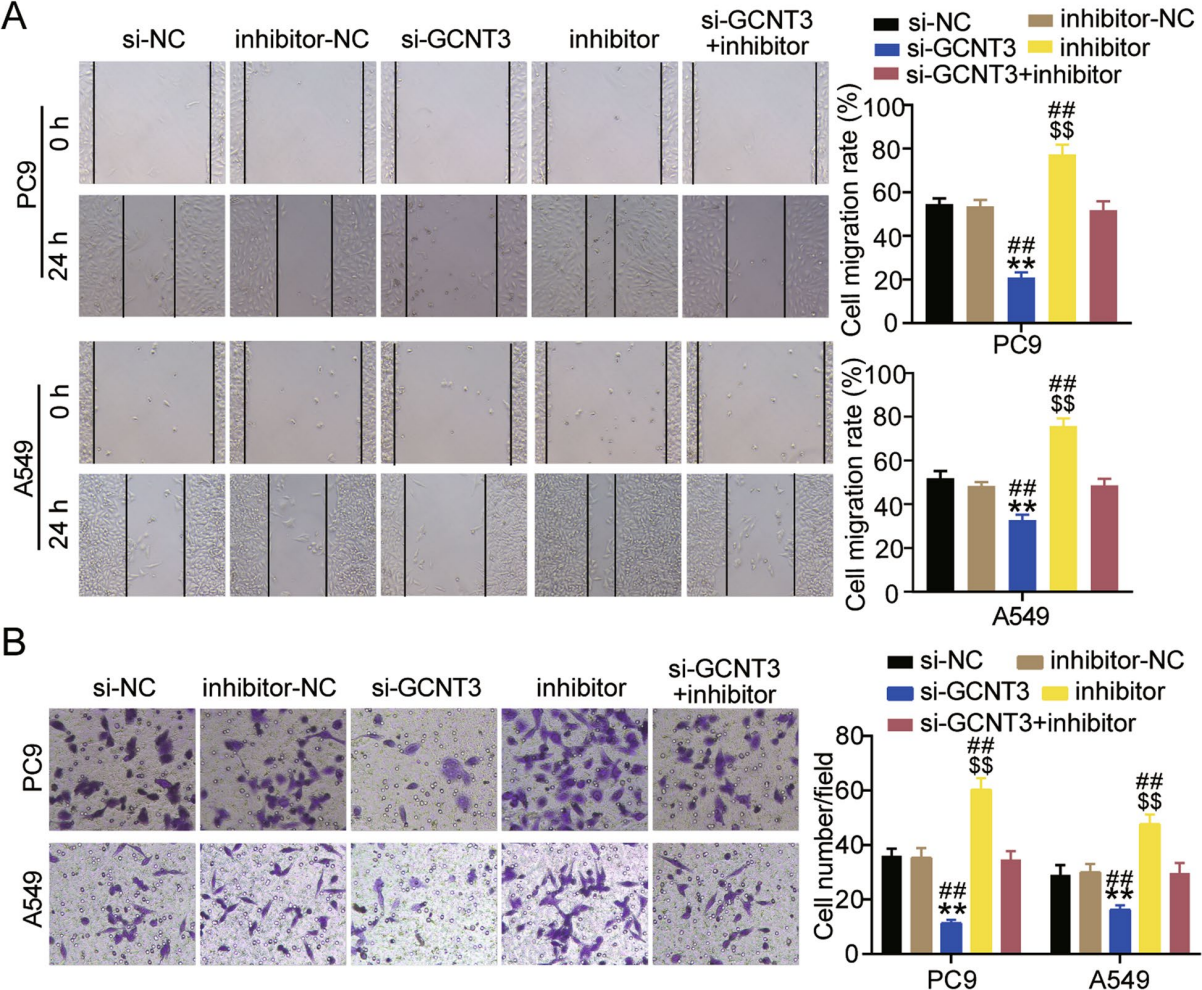
GCNT3 silencing reversed the effect of miR-195-5p on the p metastasis of LUAD cells. **A** Migration capacity was determined by wound healing assay in A549 and PC9 cells transfected with si-GCNT3 or inhibitor for 48 h. **B** Cell invasion ability was detected by transwell invasion assay in A549 and PC9 cells transfected with si-GCNT3 or inhibitor for 48 h. **P < 0.001 compared with si-NC group. $$*P* < 0.001 compared with inhibitor-NC. ##*P* < 0.001 compared with si-GCNT3 + inhibitor. si-GCNT3, siRNA of GCNT3. si-NC, negative control of siRNA. inhibitor, miR-195-5p inhibitor. inhibitor-NC, negative control of inhibitor. si-GCNT3 + inhibitor, siRNA of GCNT3 + miR-195-5p inhibitor

Next, we verified whether the effects of miR-195-5p on the biological function of LUAD cells depends on GCNT3 expression. The CCK-8 assay showed that interference with GCNT3 significantly reduced cell viability, while reversing the survival-promoting effect of miR-195-5p inhibitor (Fig. 8A). The BrdU assay confirmed that compared with the si-NC or si-GCNT3 + inhibitor groups, the cell proliferation levels of the si-GCNT3 group decreased by more than 40% (Fig. 8B). Western blotting showed that silencing GCNT3 increased Bax protein levels by more than 1.5-fold in A549 and PC9 cells, reduced Bcl-2 protein levels by more than 40%, and rescued apoptosis induced by miR-195-5p inhibitor (Fig. 8C). In addition, the wound healing assay indicated that interference with GCNT3 decreased the cell migration rate and eliminated the effect of miR-195-5p interference (Fig. 9A). The transwell assay was performed to detect cell invasion. Low expression of GCNT3 reduced the invasion levels of A549 and PC9 cells to approximately 40% compared to that of the si-NC group, as well as eliminated the cell invasion induced upon treatment with miR-195-5p inhibitor (Fig. 9B).

## Discussion

Numerous studies have revealed that LINC00511 is a multipotent cancer promoter. LINC00511 is linked with poor prognosis in breast cancer patients and facilitates the growth of cancer stem cells and tumors [35]. LINC00511 enhances proliferation, migration, and invasion, as well as restrains apoptosis of gastric and cervical cancer cells [36, 37]. In this study, we found that LINC00511 was overexpressed in LUAD, which is consistent with the results reported by Wei et al. [11]. Additionally, this study further revealed the regulation of LINC00511 on the biological function of LUAD cells, and found that LINC00511 induced the proliferation, migration, and invasion of LUAD cells and inhibited apoptosis. This finding is also consistent with observations in gastric cancer [36] and cervical cancer [37]. This suggests that LINC00511 may be a tumor-promoting factor in LUAD.

Accumulated literature indicates that miR-195-5p serves as a tumor-inhibiting factor in a variety of cancers, including colorectal cancer [38], breast cancer [39], and cervical cancer [40]. Moreover, Long et al. [33] found that miR-195-5p was downregulated in lung cancer, inhibiting the survival and metastasis of cells. Xin et al. [34] also revealed a decrease in miR-195-5p expression in LUAD, which inhibits malignant cell proliferation. In this study, it was revealed that silencing miR-195-5p had a positive regulatory effect on the proliferation, migration, and invasion of LUAD cells, and a negative regulatory effect on apoptosis, which is in agreement with the results of previous studies. In addition, bioinformatics analysis, luciferase assay, and RIP analysis indicated that miR-195-5p was mediated by LINC00511, which was consistent with the observation that miR-195-5p binds to LINC00511 as reported by Li et al. [41].

Previous studies found that GCNT3 was significantly correlated with clinicopathological malignant phenotype and poor overall survival of lung cancer patients, and GCNT3 knockout inhibited epithelial mesenchymal transformation, invasion, migration, and proliferation ability of lung cancer cells [19]. Clinically, this study also showed an increase in GCNT3 levels in LUAD. Functional analysis also showed that downregulating the expression of GCNT3 could attenuate the proliferation, migration, and invasion of LUAD cells. In addition, further exploration showed that GCNT3 was a target gene of miR-195-5p and was involved in altering the function of miR-195-5p in LAUD cells. Taken together, these analyses revealed that GCNT3 was regulated by LINC00511/miR-195-5p to maintain the malignant proliferation of LAUD cells.

However, it is not sufficient to demonstrate the impact of LINC00511/miR-195-5p/GCNT3 in LUAD at the cellular level. Elucidating the mechanism by which LINC00511 affects the metastasis of cancer cells *in vivo* is of great value to further understand the progression of LUAD. In addition, analysis of the correlation of pathological phenotypes in a larger sample size is the focus of future research.

## Conclusions

In conclusion, we elucidated that miR-195-5p binds to LINC00511 and regulates the target gene GCNT3 in LUAD. These data indicate a molecular pathway mediated by LINC00511 in the progression of LUAD. In addition, miR-195-5p/GCNT3 acts as a potential target for LINC00511 and is of great significance in the diagnosis and treatment of LUAD.

## Supplementary Information

**Additional file 1.**

## Data Availability

All data generated or analysed during this study can be found in below websites. One mRNA microarrays (GSE85841) from GEO DataSets (https://www.ncbi.nlm.nih.gov/gds) were used to screen the upregulated genes. The expression of the 5 genes in LUAD and LUSC. Data obtained from GEPIA database (http://gepia.cancer-pku.cn/). starBase (http://starbase.info/) was used to predict the binding sites.

## Abbreviations

ceRNA: competitive endogenous RNA
lncRNA: long non-coding RNA
LUAD: lung adenocarcinoma
miRNAs: microRNAs
