# Supplementary figures and images for: LINC00511 enhances LUAD malignancy by upregulating GCNT3 via miR-195-5p
Source: BMC Cancer. 2022 Apr 10;22:389. doi: 10.1186/s12885-022-09459-7 (PMC8994914; doi:10.1186/s12885-022-09459-7)

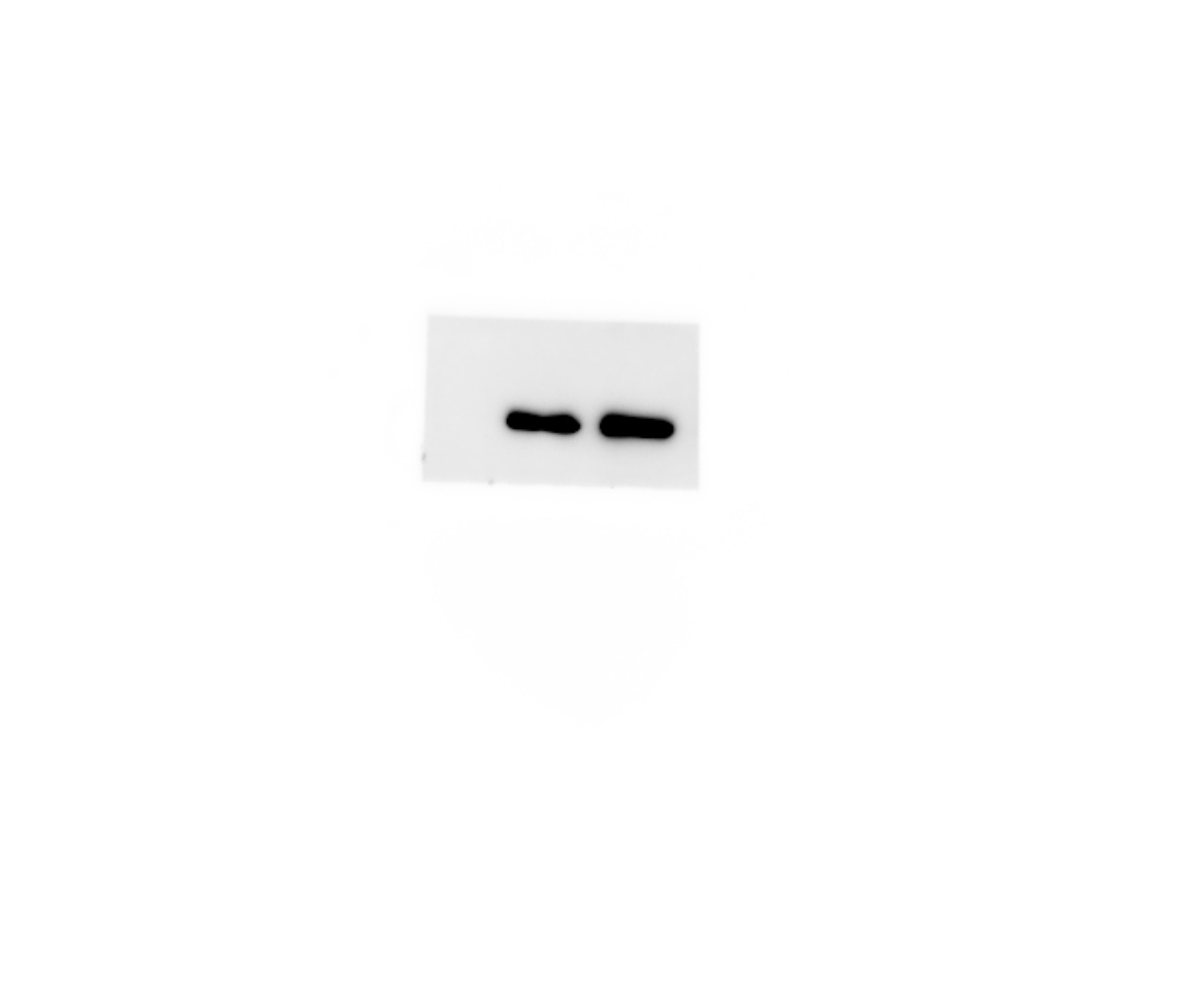

Supplement: Supplementary file 1 — Additional file 1. [file 12885_2022_9459_MOESM1_ESM.zip › Supplementary Information/WB/Figure 3C A549 GAPDH.jpg]

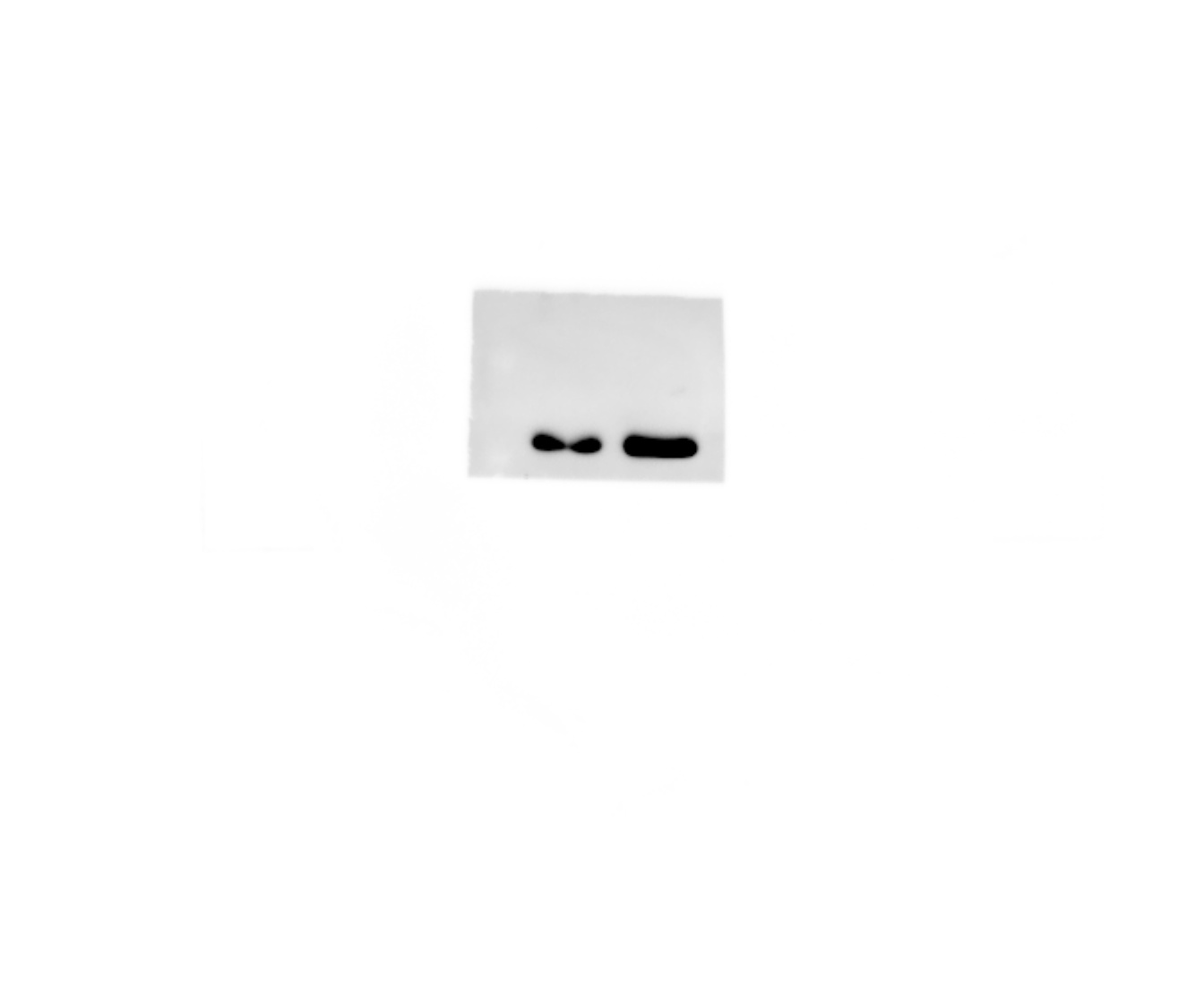

Supplement: Supplementary file 1 — Additional file 1. [file 12885_2022_9459_MOESM1_ESM.zip › Supplementary Information/WB/Figure 3C A549 bax.jpg]

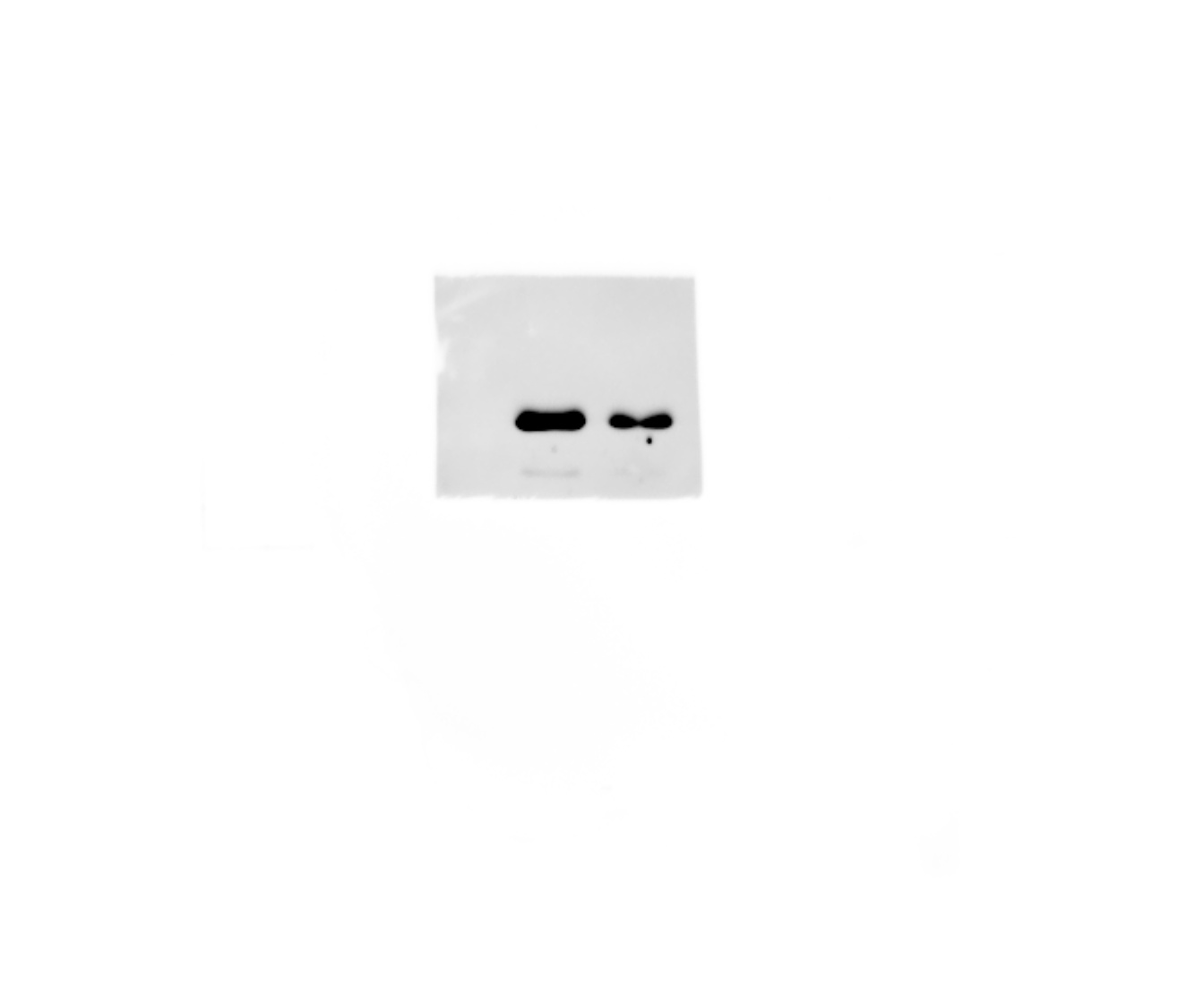

Supplement: Supplementary file 1 — Additional file 1. [file 12885_2022_9459_MOESM1_ESM.zip › Supplementary Information/WB/Figure 3C A549 bcl-2.jpg]

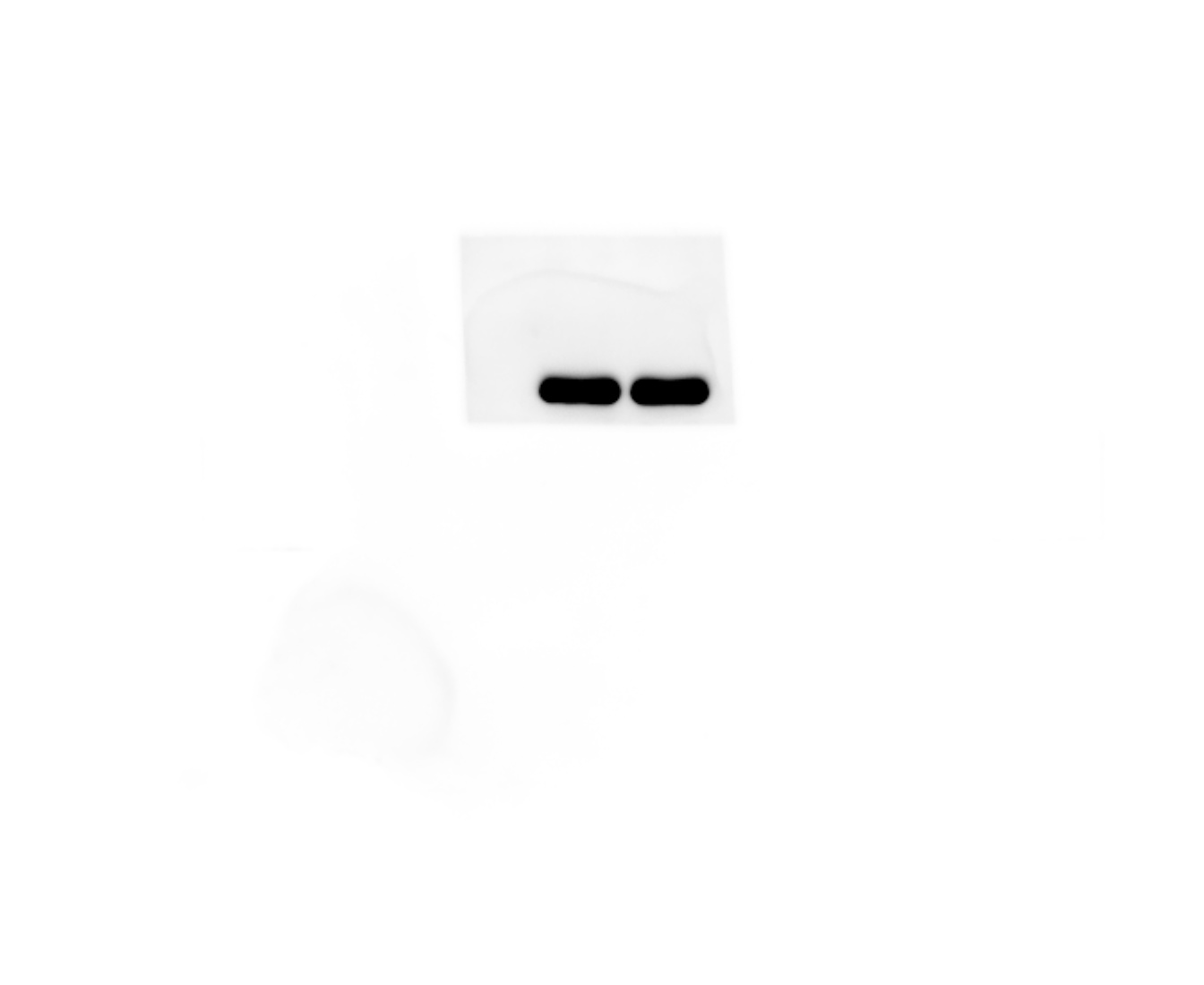

Supplement: Supplementary file 1 — Additional file 1. [file 12885_2022_9459_MOESM1_ESM.zip › Supplementary Information/WB/Figure 3C PC9 GAPDH.jpg]

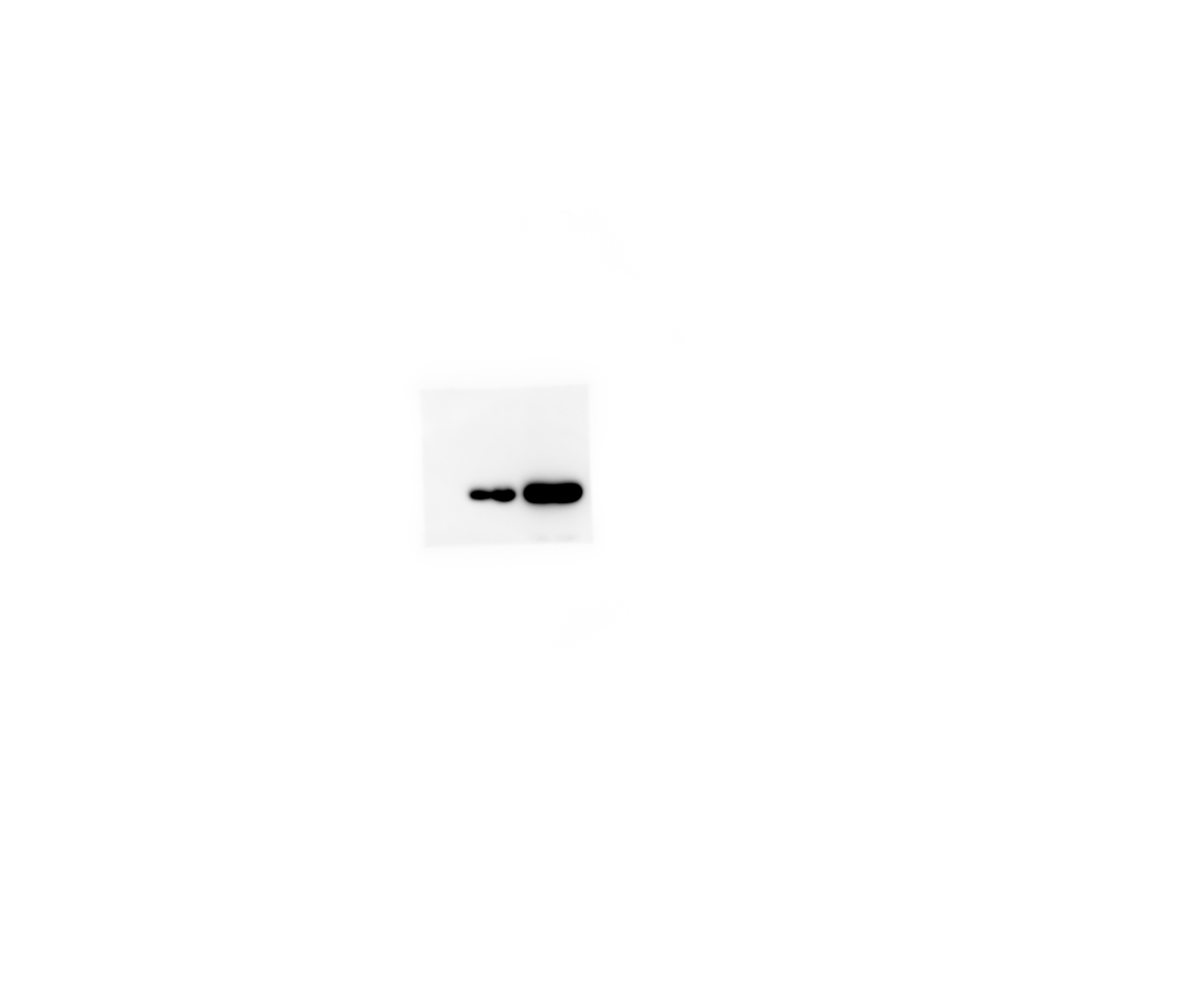

Supplement: Supplementary file 1 — Additional file 1. [file 12885_2022_9459_MOESM1_ESM.zip › Supplementary Information/WB/Figure 3C PC9 bax.jpg]

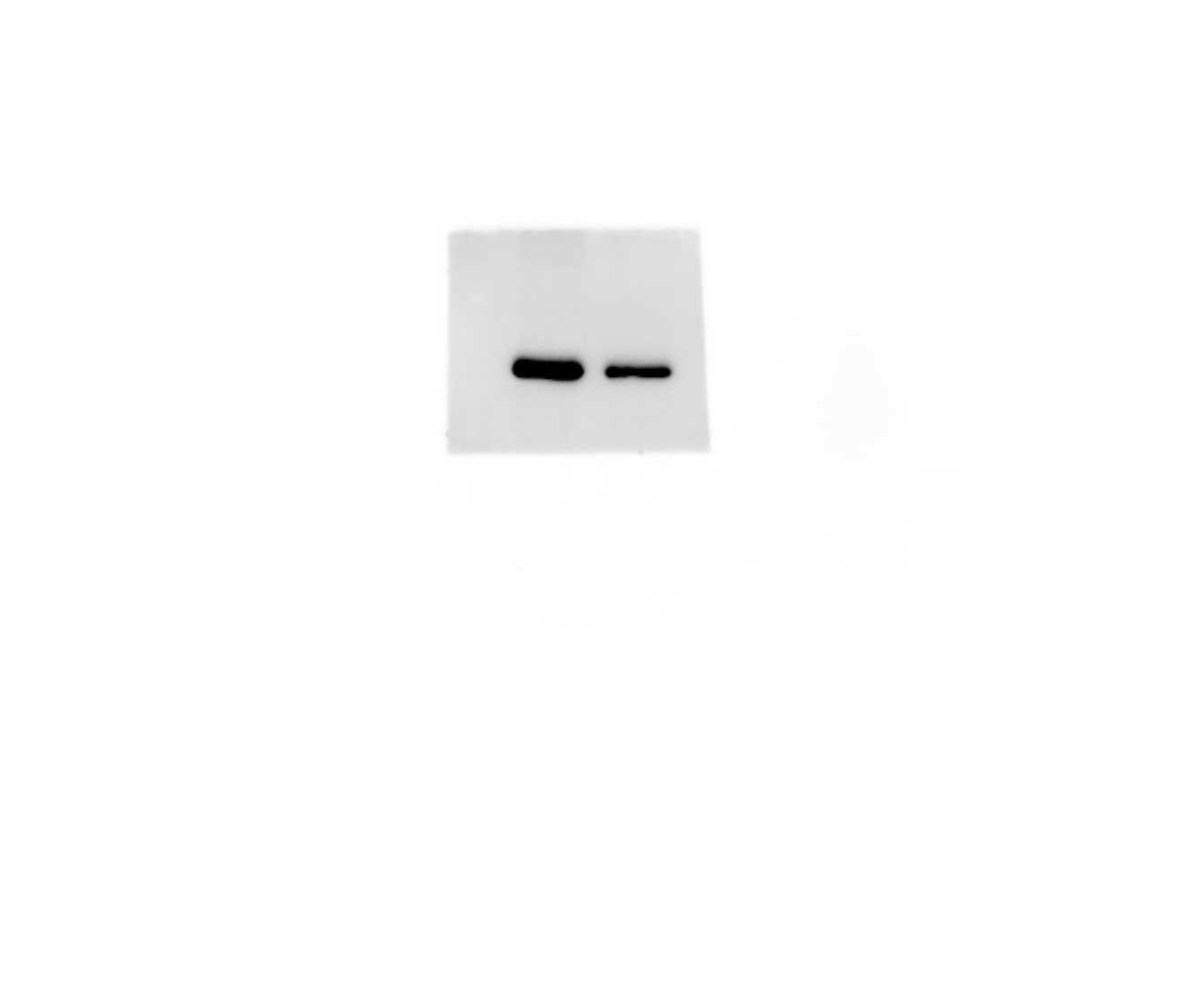

Supplement: Supplementary file 1 — Additional file 1. [file 12885_2022_9459_MOESM1_ESM.zip › Supplementary Information/WB/Figure 3C PC9 bcl-2.jpg]

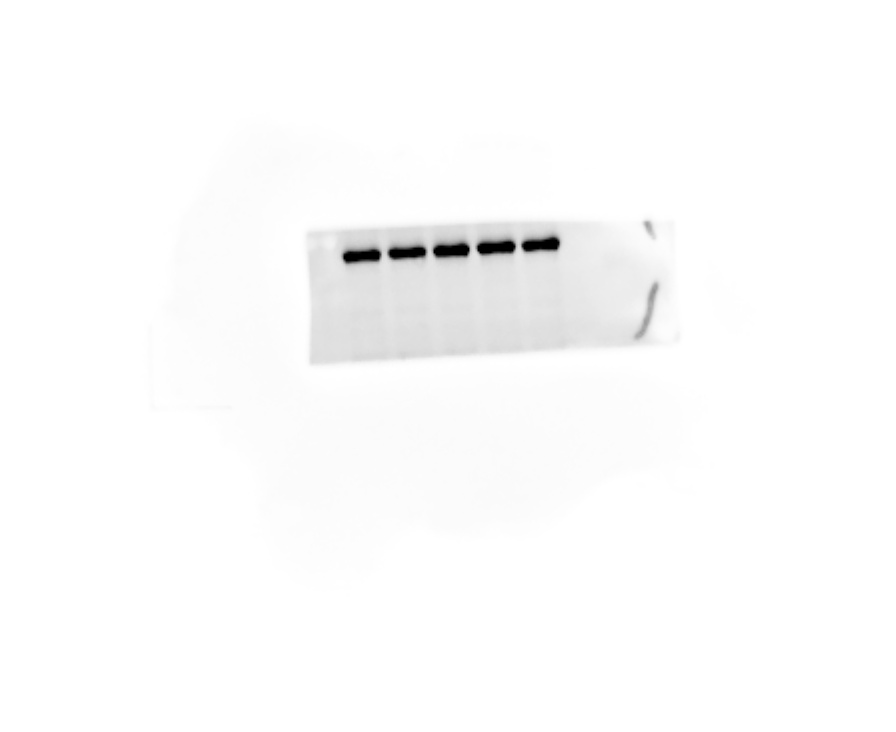

Supplement: Supplementary file 1 — Additional file 1. [file 12885_2022_9459_MOESM1_ESM.zip › Supplementary Information/WB/Figure 5C A549 GAPDH.jpg]

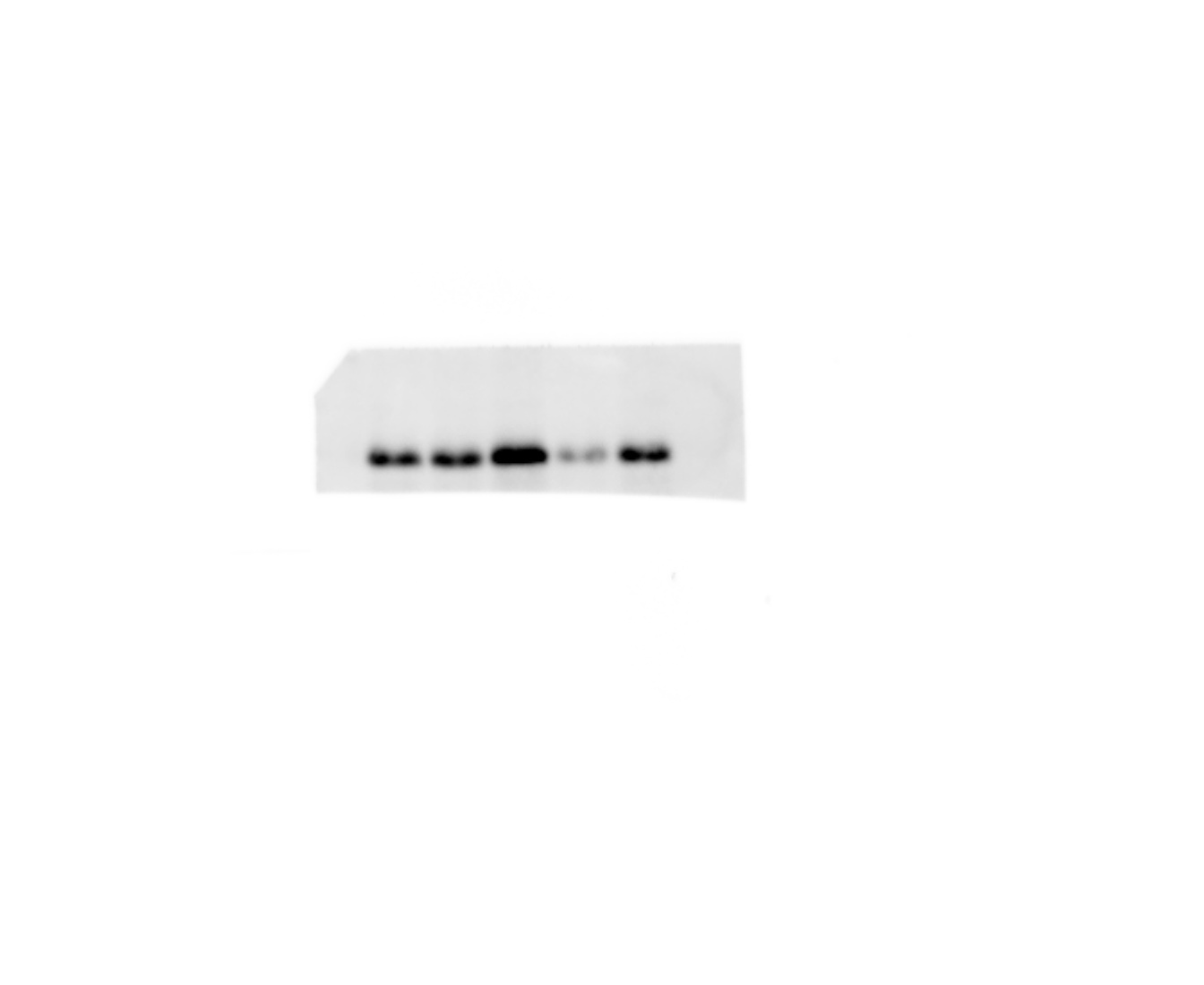

Supplement: Supplementary file 1 — Additional file 1. [file 12885_2022_9459_MOESM1_ESM.zip › Supplementary Information/WB/Figure 5C A549 bax.jpg]

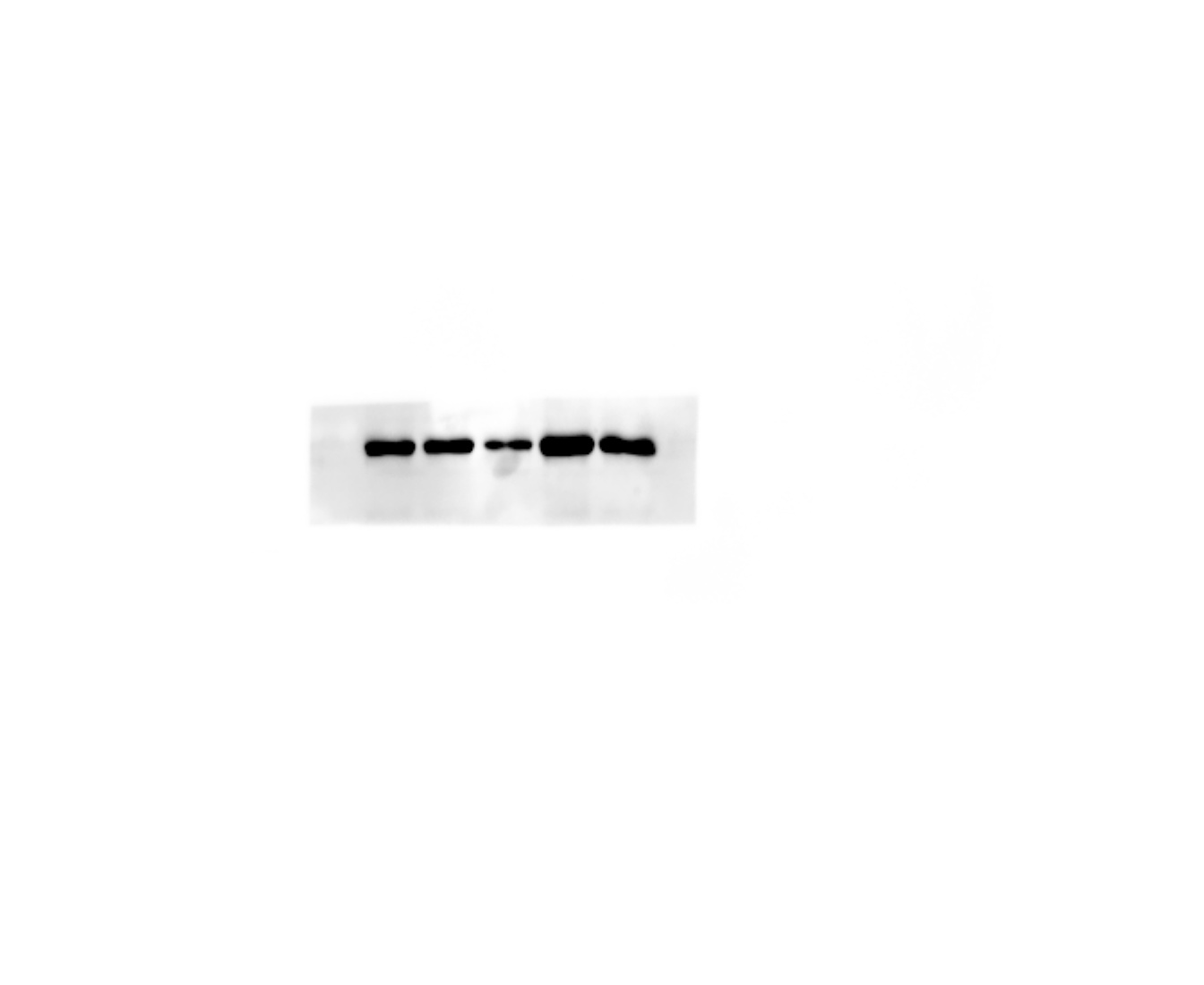

Supplement: Supplementary file 1 — Additional file 1. [file 12885_2022_9459_MOESM1_ESM.zip › Supplementary Information/WB/Figure 5C A549 bcl-2.jpg]

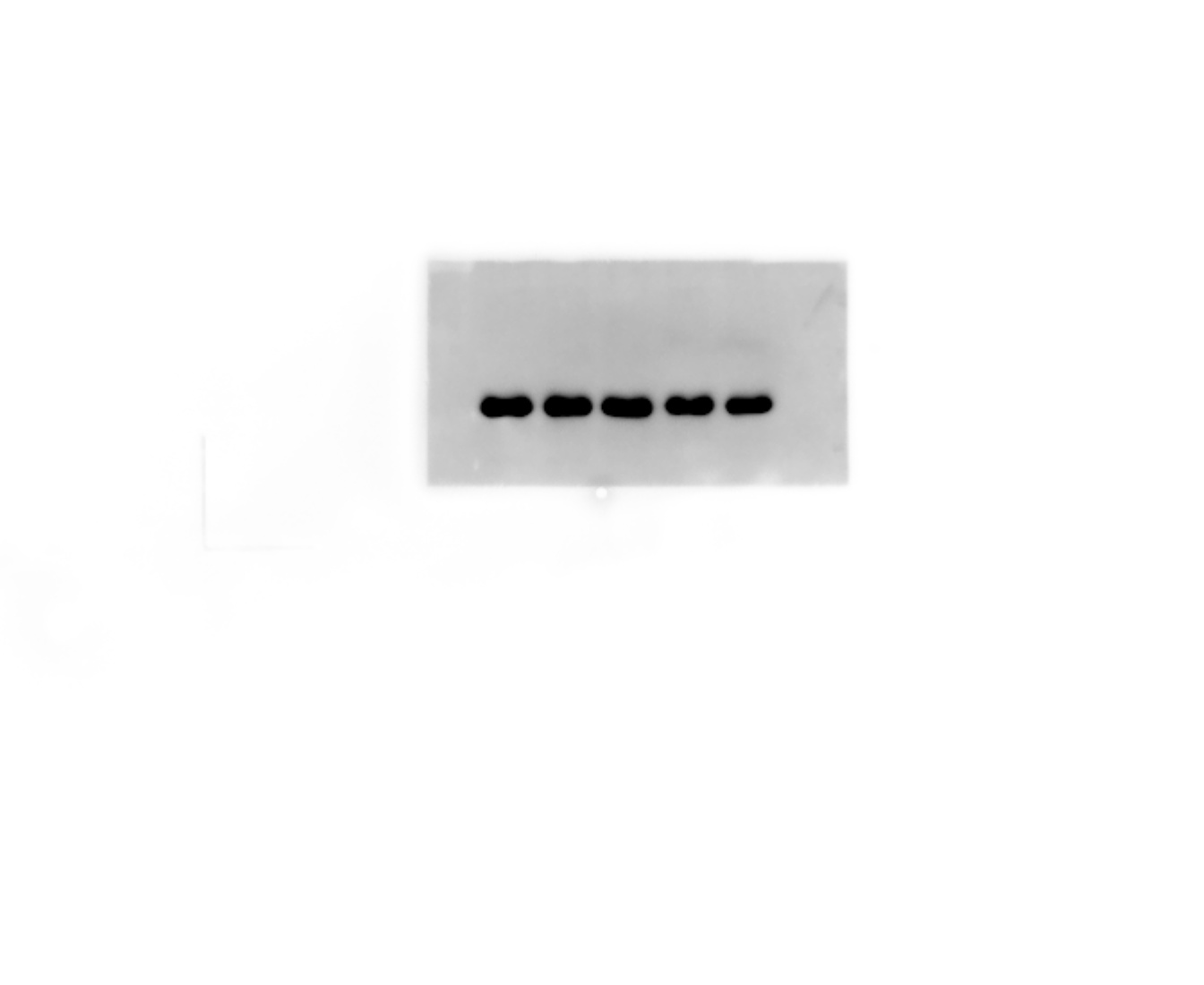

Supplement: Supplementary file 1 — Additional file 1. [file 12885_2022_9459_MOESM1_ESM.zip › Supplementary Information/WB/Figure 5C PC9 GAPDH.jpg]

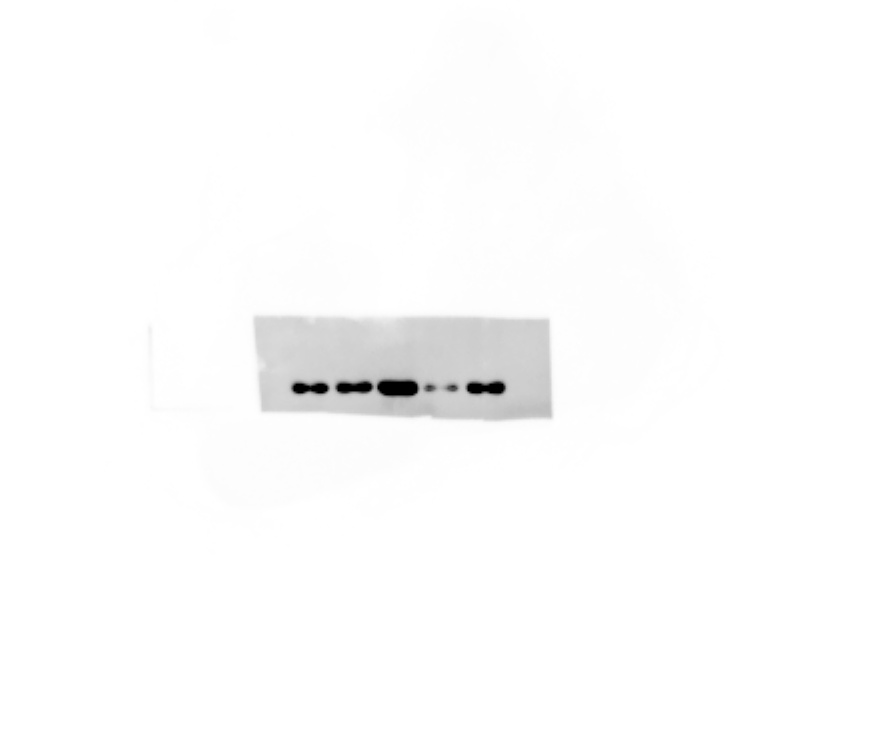

Supplement: Supplementary file 1 — Additional file 1. [file 12885_2022_9459_MOESM1_ESM.zip › Supplementary Information/WB/Figure 5C PC9 bax.jpg]

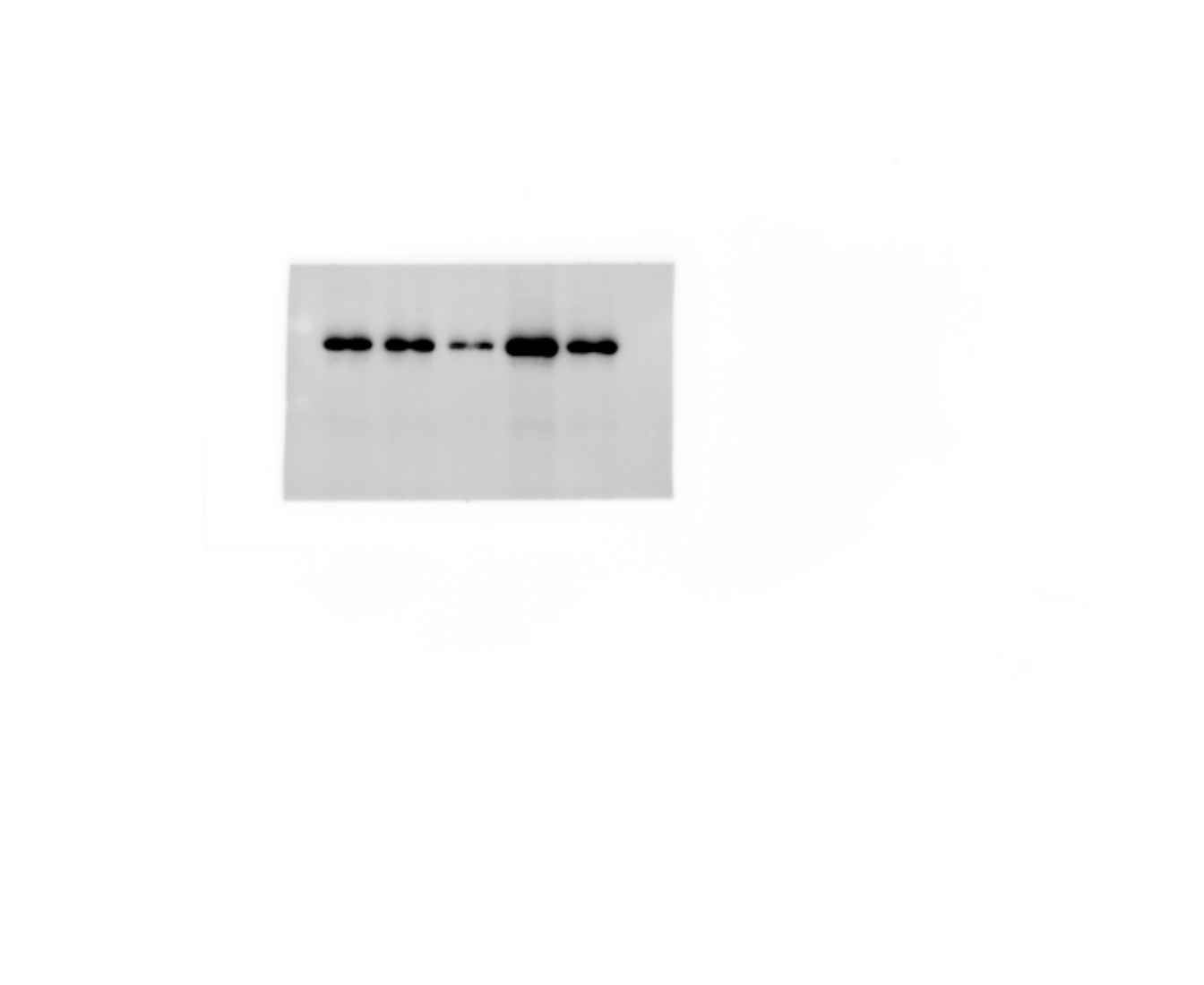

Supplement: Supplementary file 1 — Additional file 1. [file 12885_2022_9459_MOESM1_ESM.zip › Supplementary Information/WB/Figure 5C PC9 bcl-2.jpg]

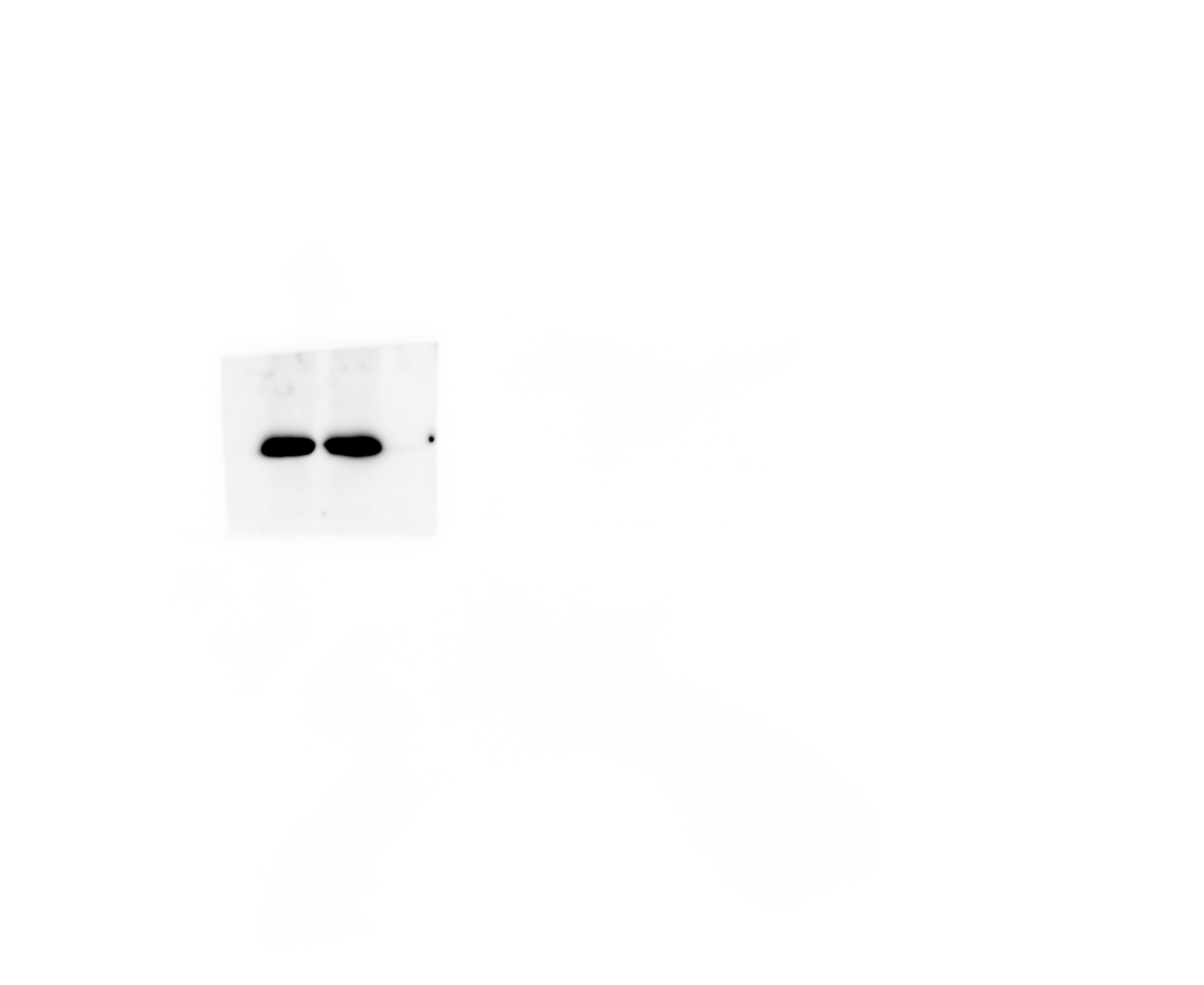

Supplement: Supplementary file 1 — Additional file 1. [file 12885_2022_9459_MOESM1_ESM.zip › Supplementary Information/WB/Figure 7G A549 GAPDH.jpg]

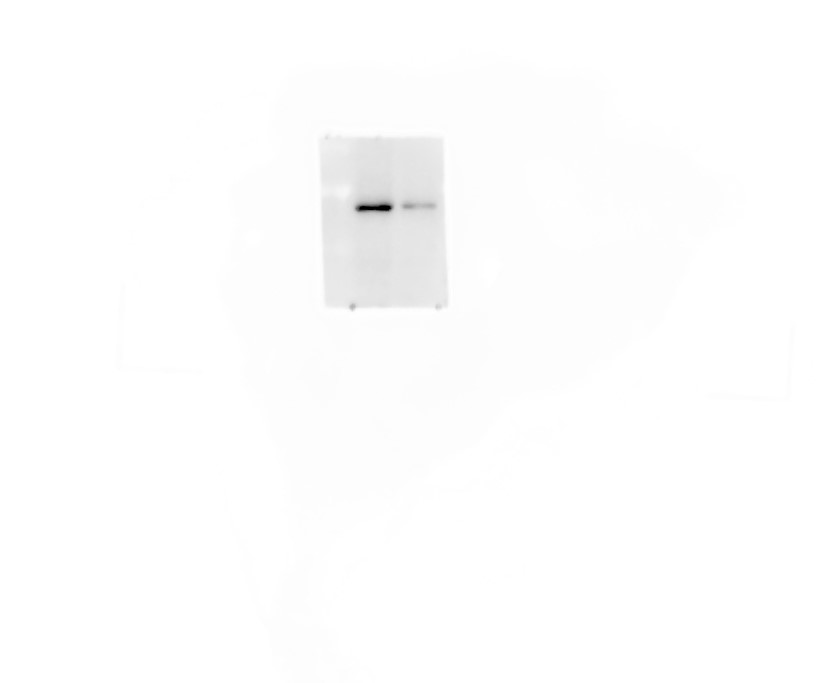

Supplement: Supplementary file 1 — Additional file 1. [file 12885_2022_9459_MOESM1_ESM.zip › Supplementary Information/WB/Figure 7G A549 GCNT3.jpg]

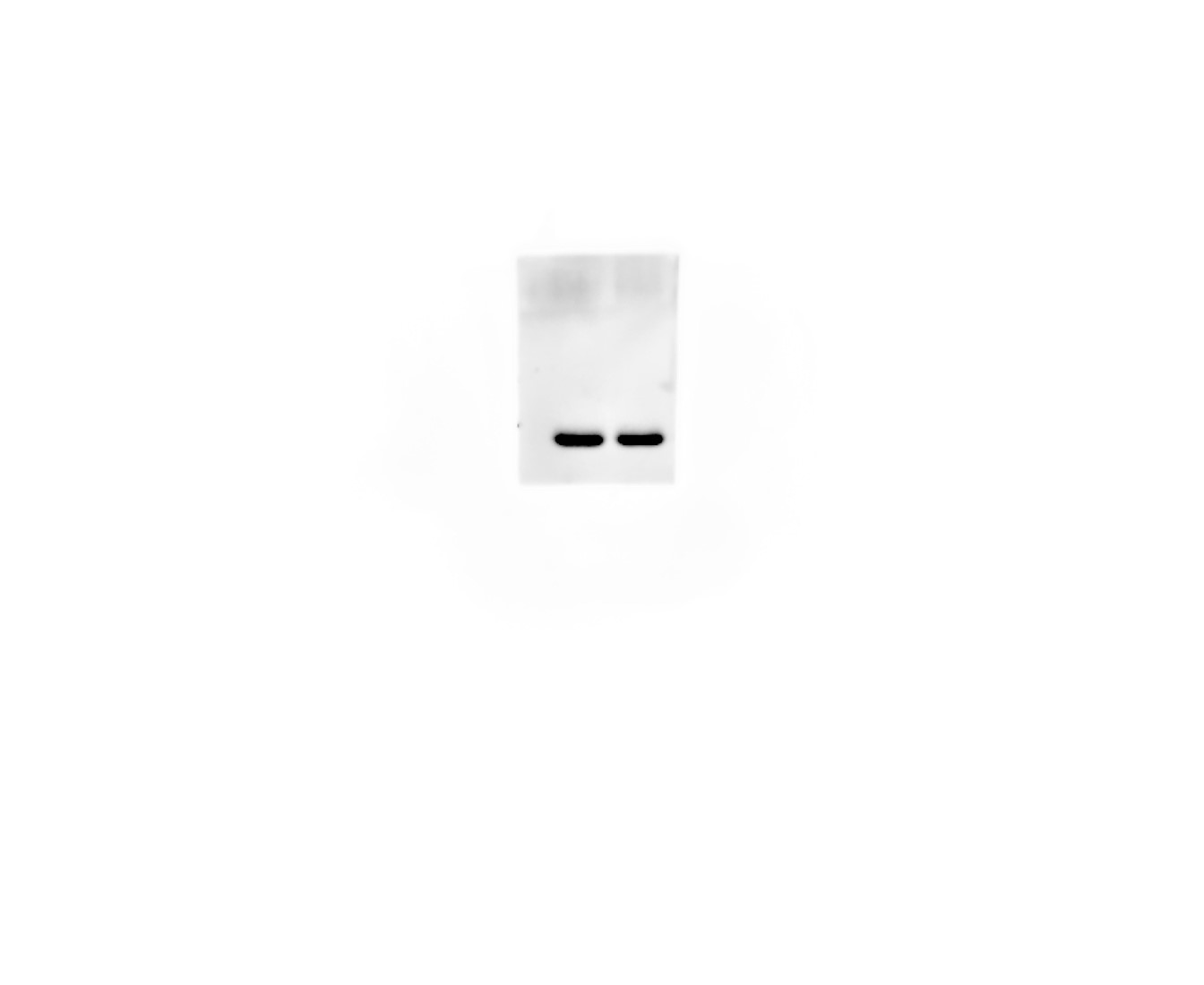

Supplement: Supplementary file 1 — Additional file 1. [file 12885_2022_9459_MOESM1_ESM.zip › Supplementary Information/WB/Figure 7G PC9 GAPDH.jpg]

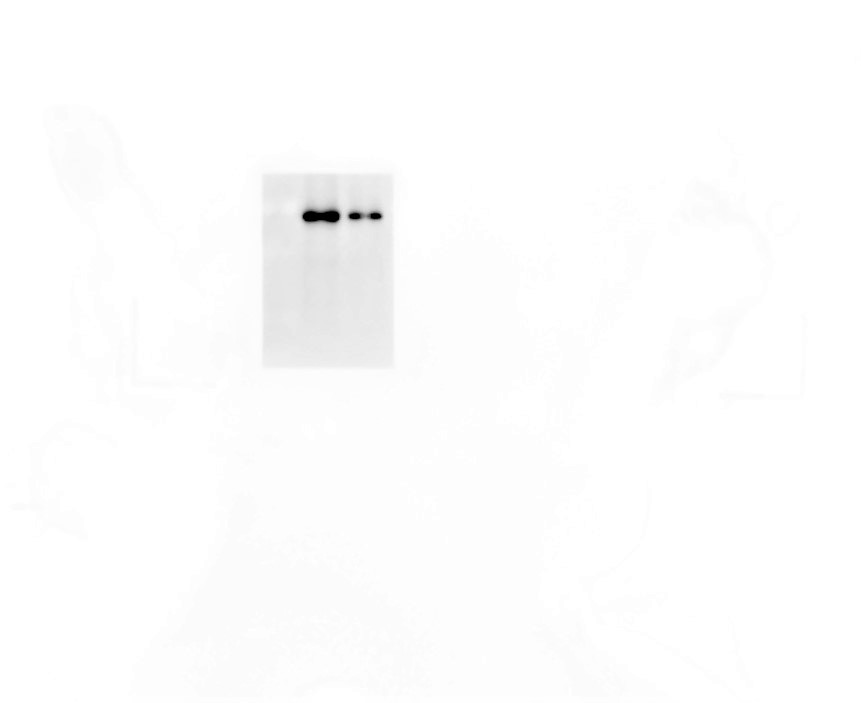

Supplement: Supplementary file 1 — Additional file 1. [file 12885_2022_9459_MOESM1_ESM.zip › Supplementary Information/WB/Figure 7G PC9 GCNT3.jpg]

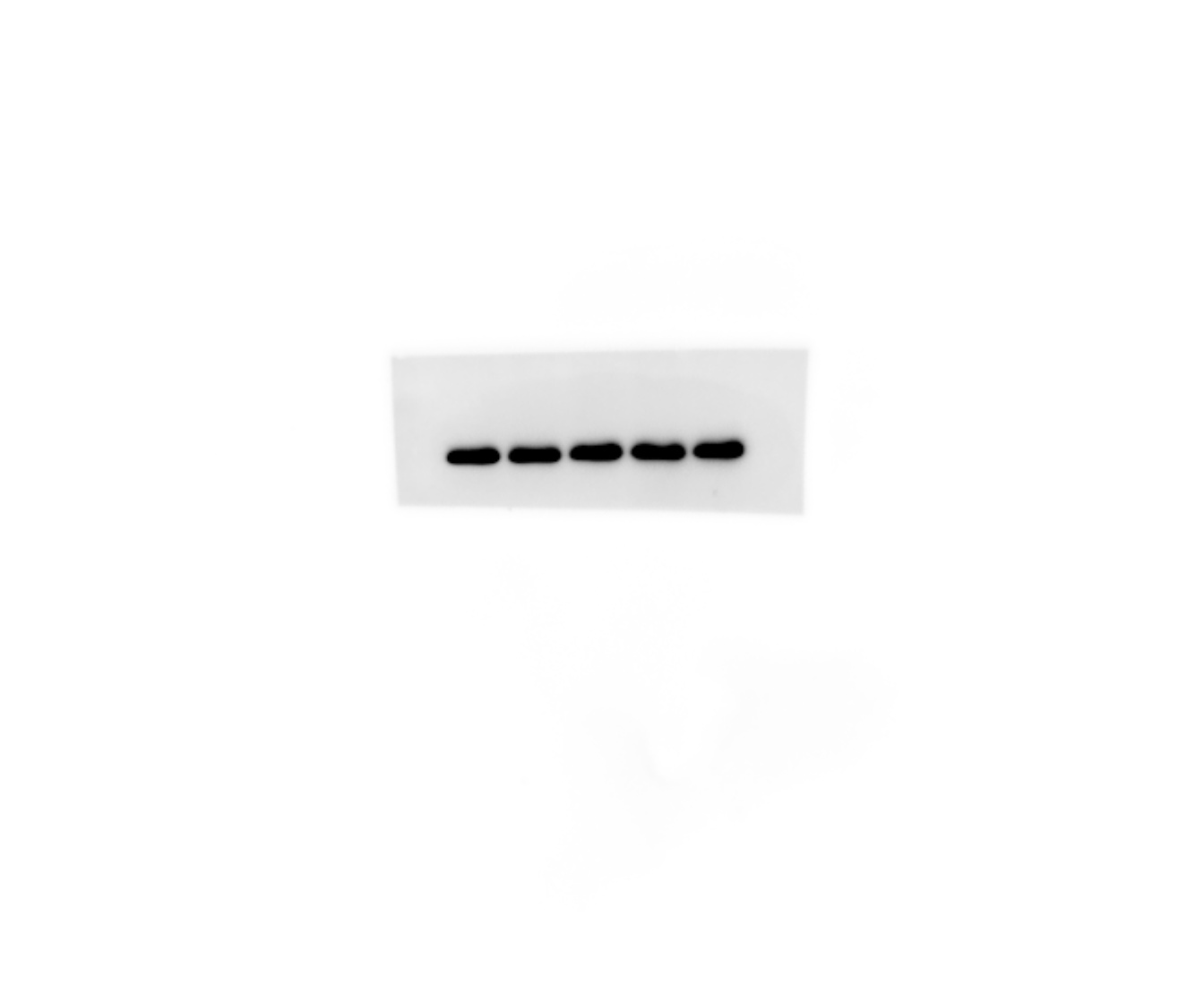

Supplement: Supplementary file 1 — Additional file 1. [file 12885_2022_9459_MOESM1_ESM.zip › Supplementary Information/WB/Figure 7H A549 GAPDH.jpg]

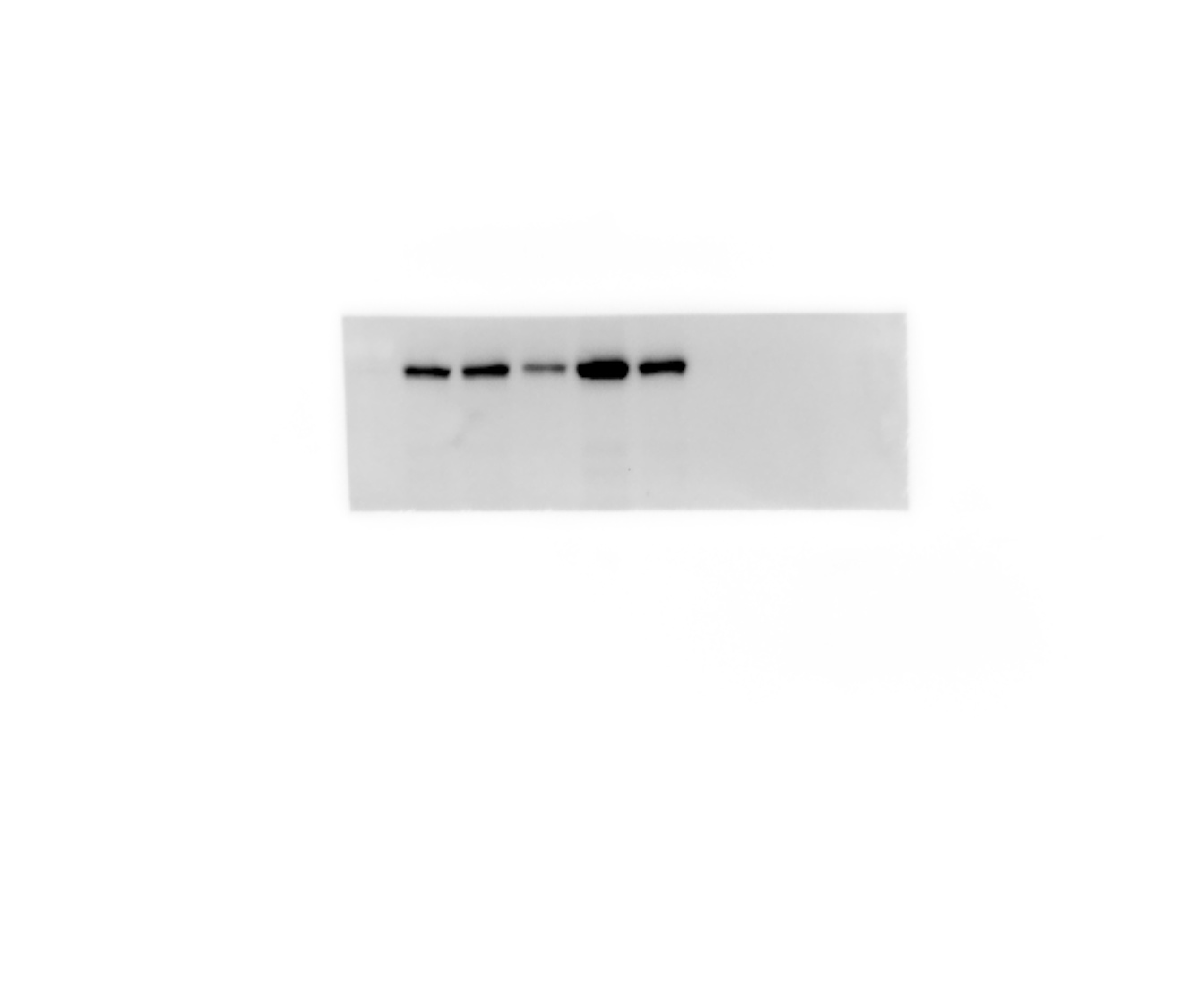

Supplement: Supplementary file 1 — Additional file 1. [file 12885_2022_9459_MOESM1_ESM.zip › Supplementary Information/WB/Figure 7H A549 GCNT3.jpg]

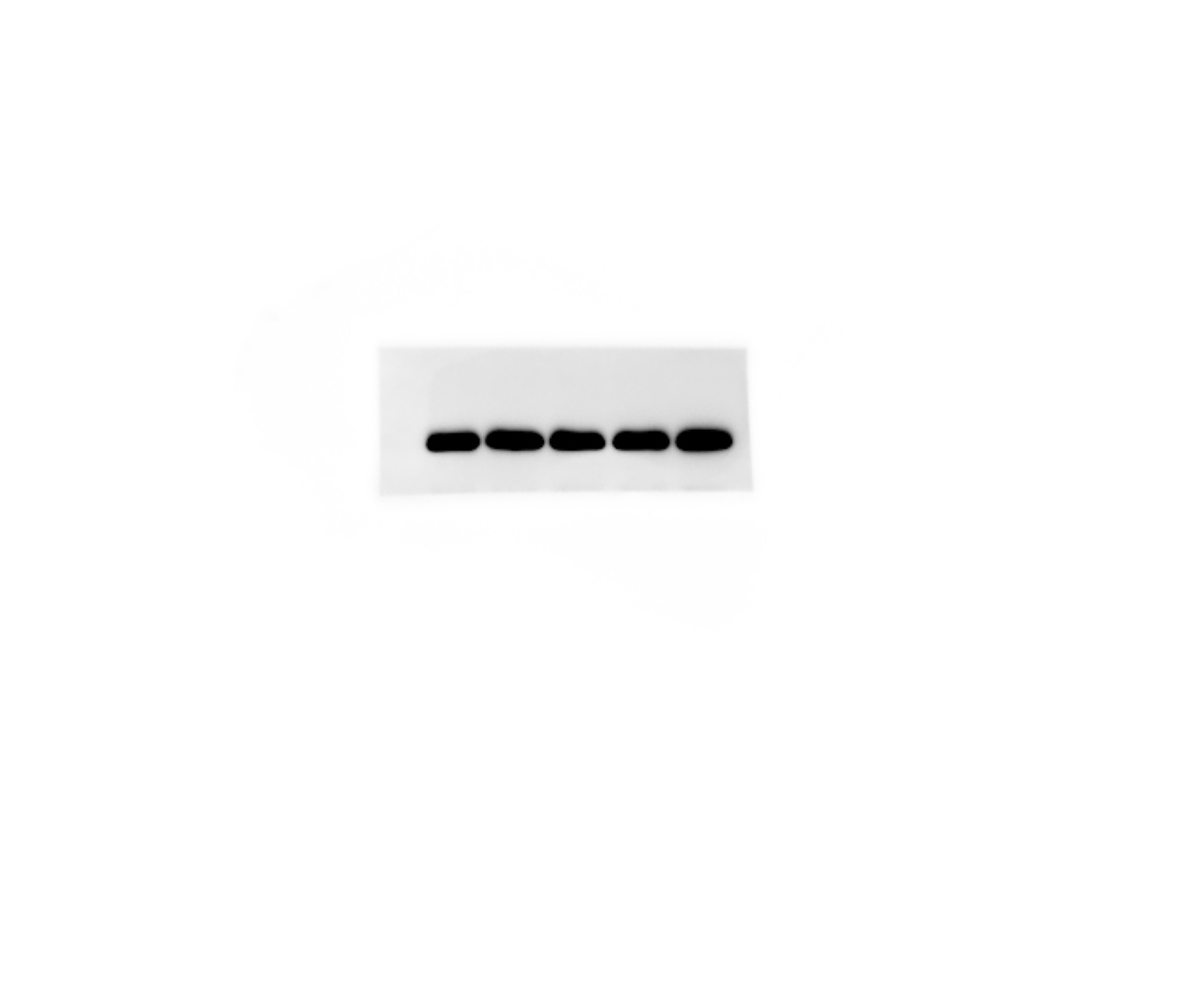

Supplement: Supplementary file 1 — Additional file 1. [file 12885_2022_9459_MOESM1_ESM.zip › Supplementary Information/WB/Figure 7H PC9 GAPDH.jpg]

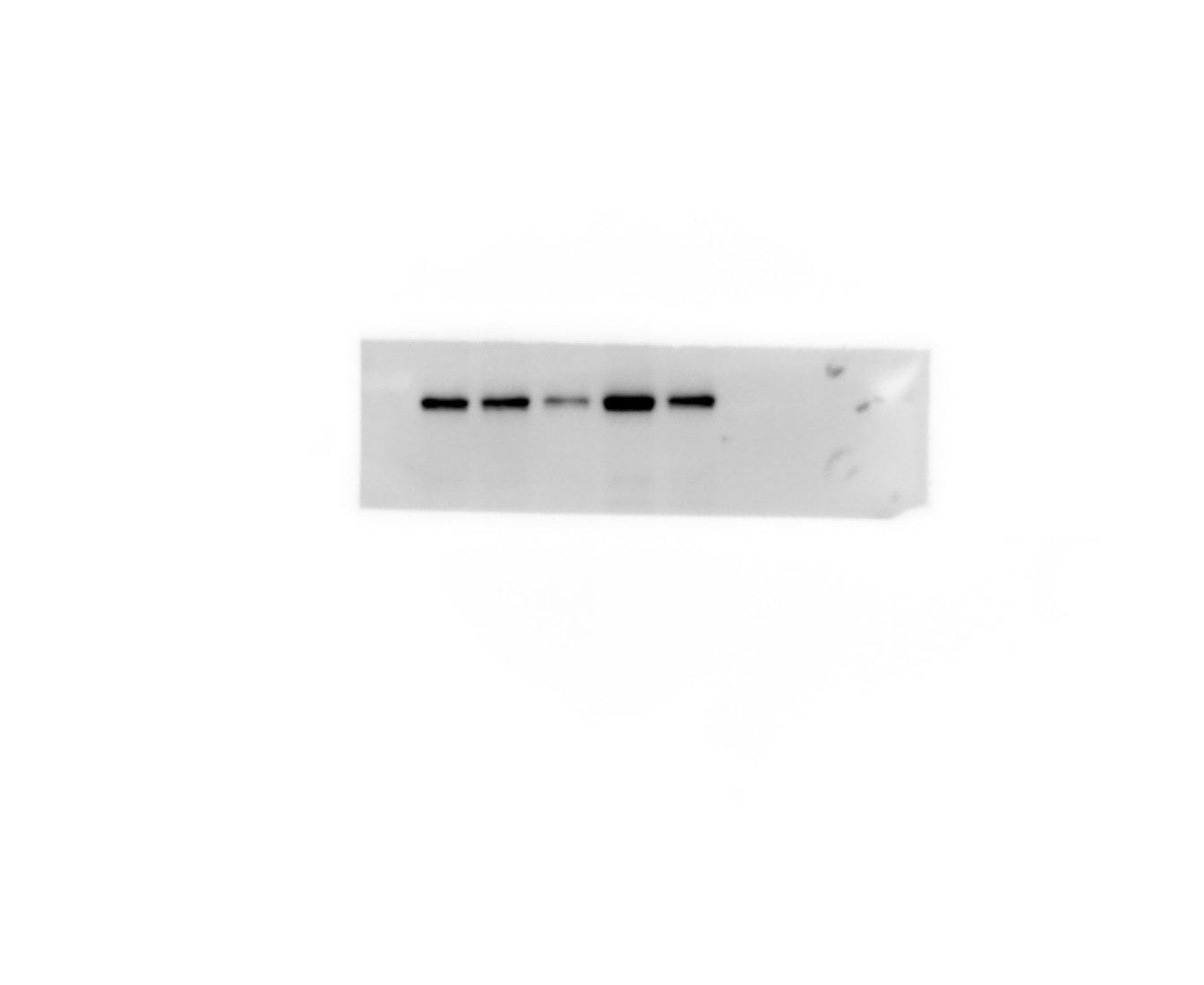

Supplement: Supplementary file 1 — Additional file 1. [file 12885_2022_9459_MOESM1_ESM.zip › Supplementary Information/WB/Figure 7H PC9 GCNT3.jpg]

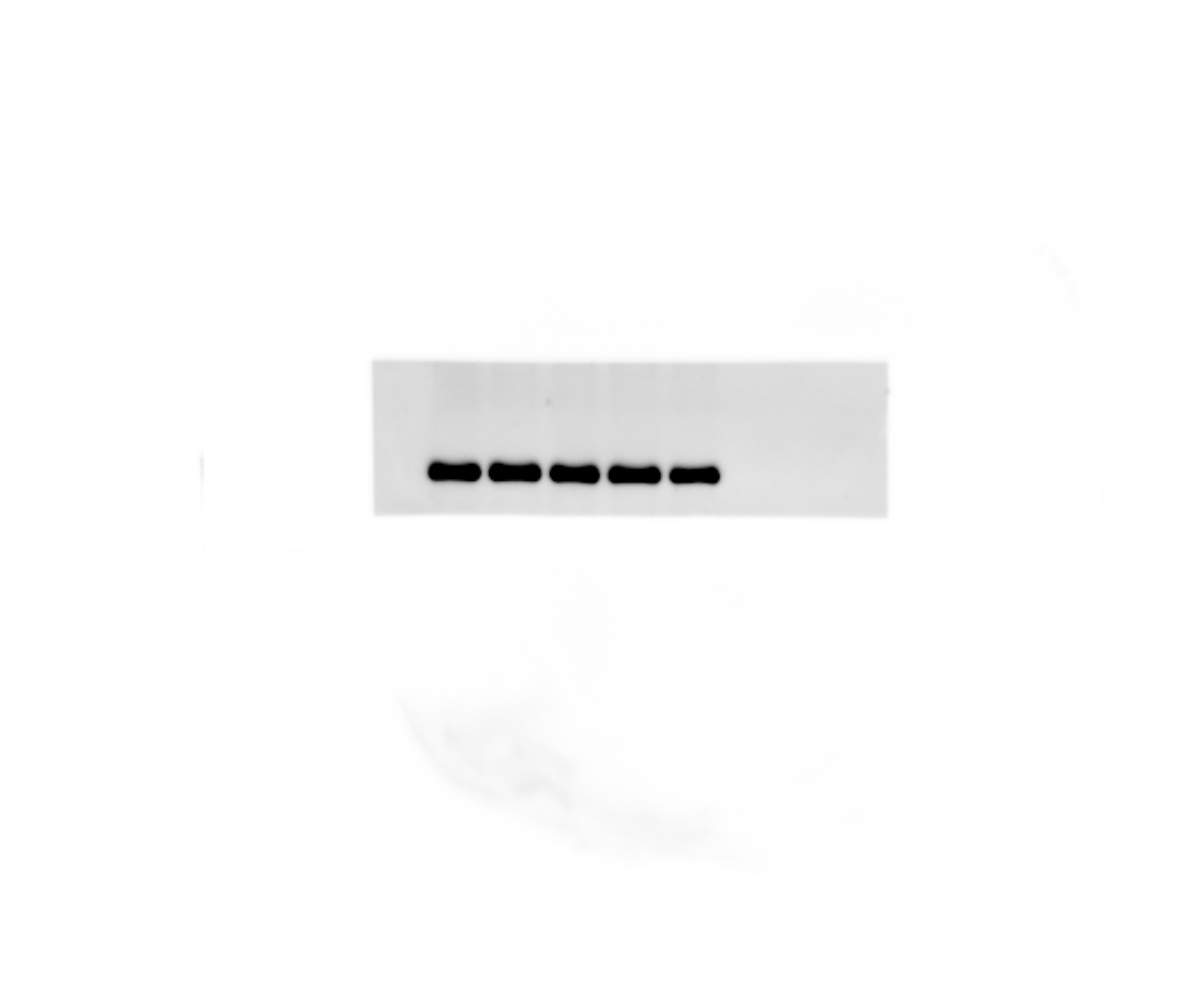

Supplement: Supplementary file 1 — Additional file 1. [file 12885_2022_9459_MOESM1_ESM.zip › Supplementary Information/WB/Figure 8C A549 GAPDH.jpg]

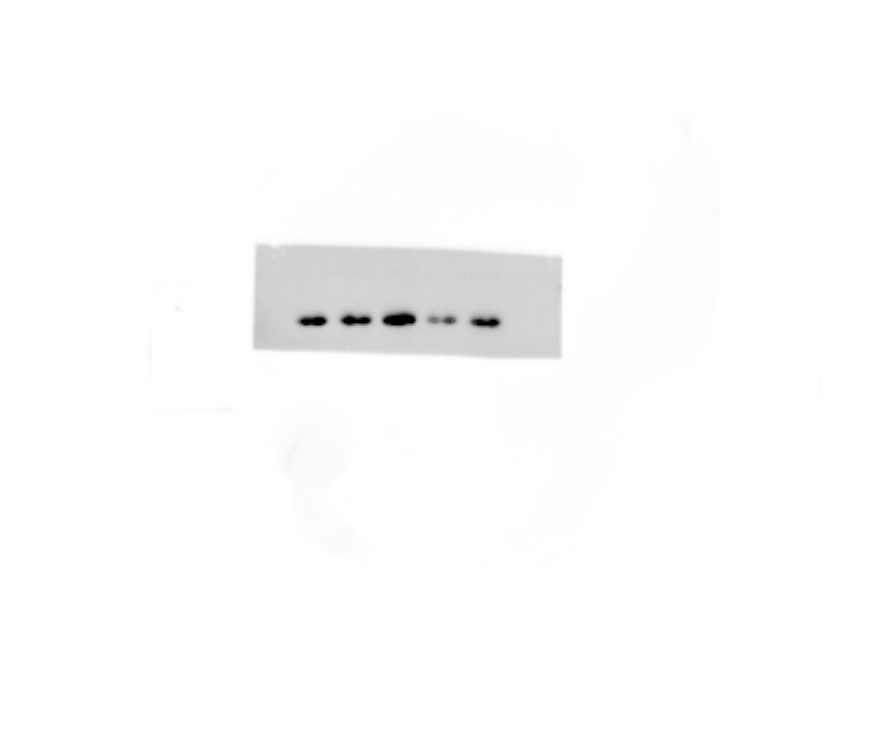

Supplement: Supplementary file 1 — Additional file 1. [file 12885_2022_9459_MOESM1_ESM.zip › Supplementary Information/WB/Figure 8C A549 bax.jpg]

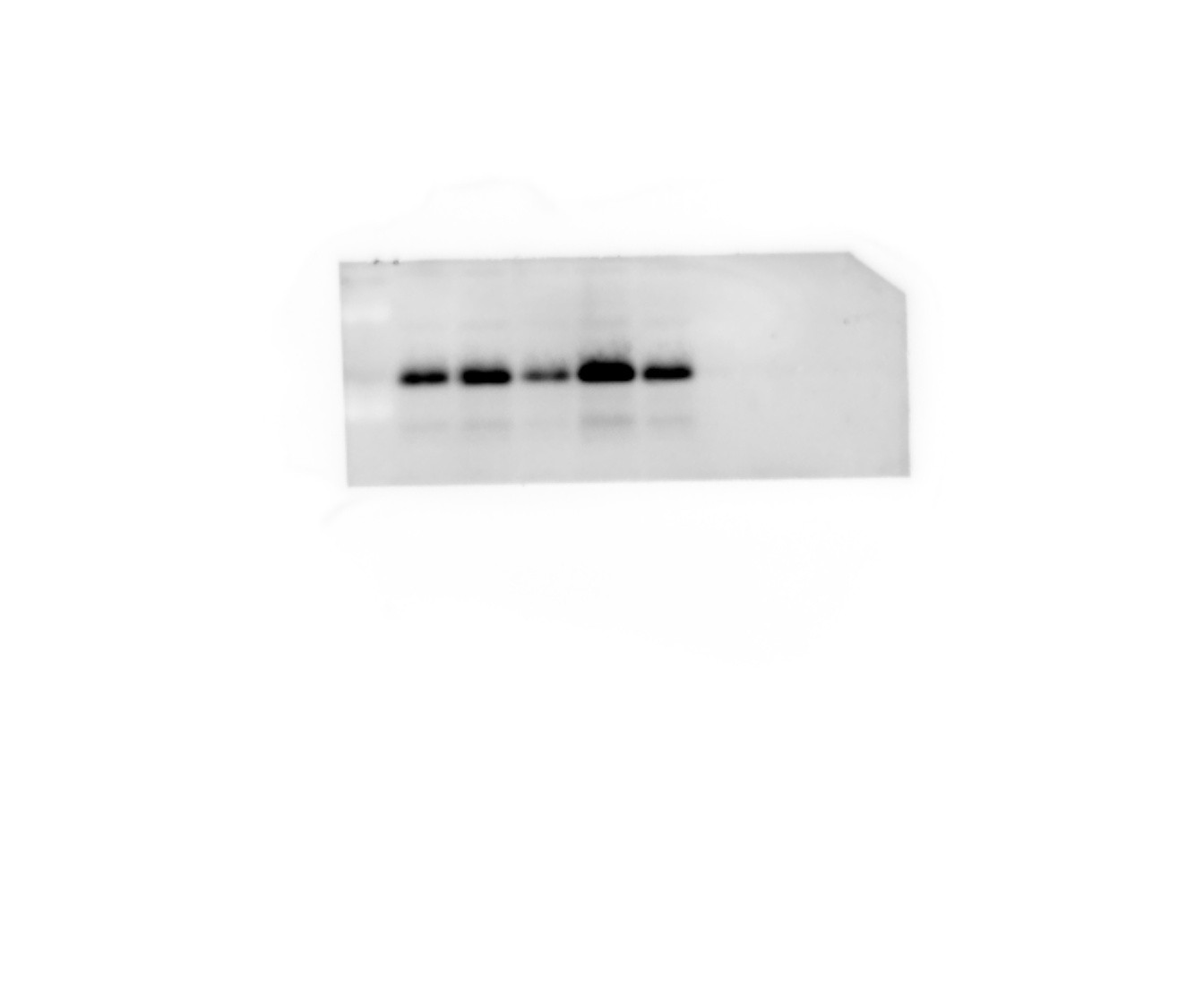

Supplement: Supplementary file 1 — Additional file 1. [file 12885_2022_9459_MOESM1_ESM.zip › Supplementary Information/WB/Figure 8C A549 bcl-2.jpg]

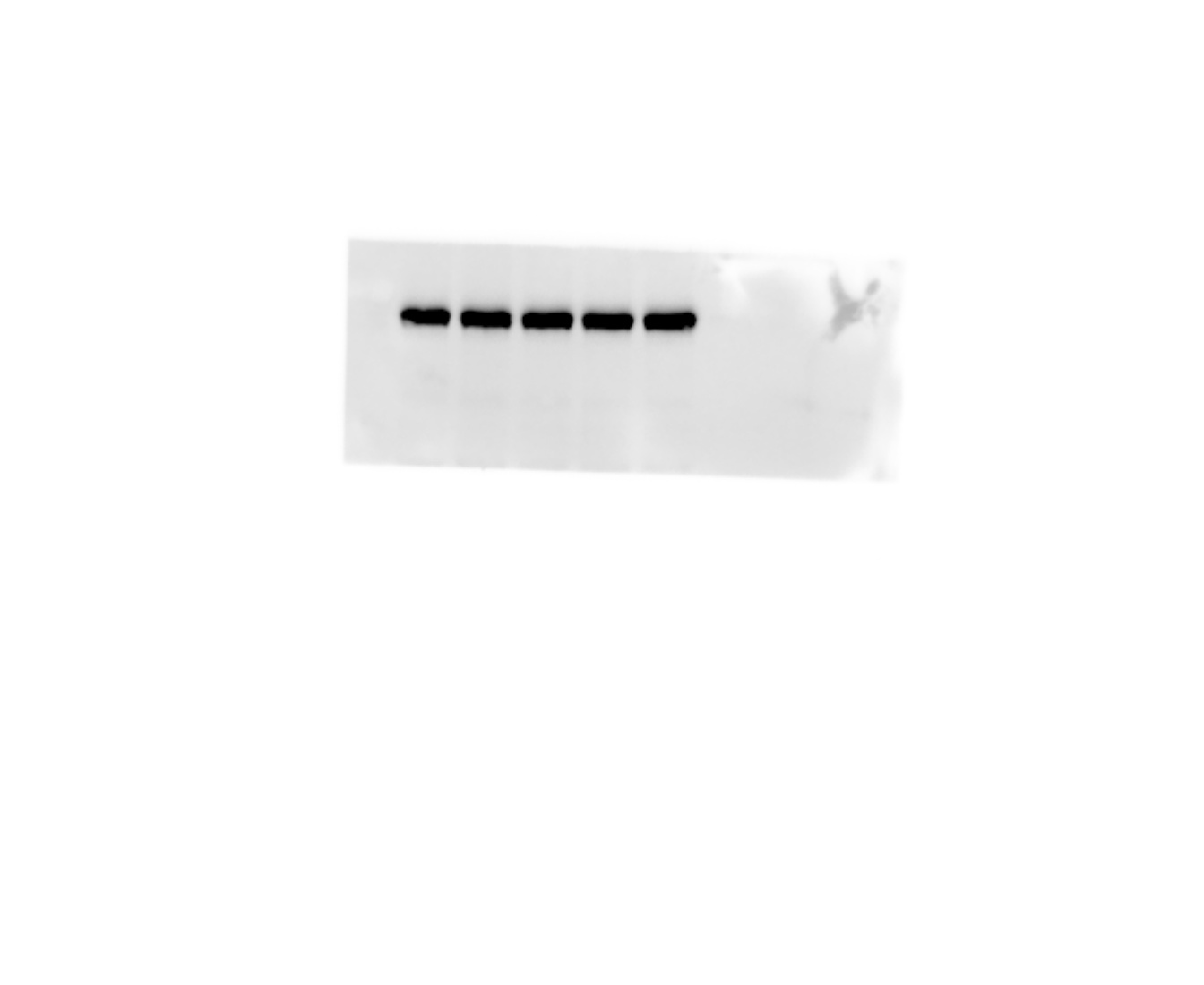

Supplement: Supplementary file 1 — Additional file 1. [file 12885_2022_9459_MOESM1_ESM.zip › Supplementary Information/WB/Figure 8C PC9 GAPDH.jpg]

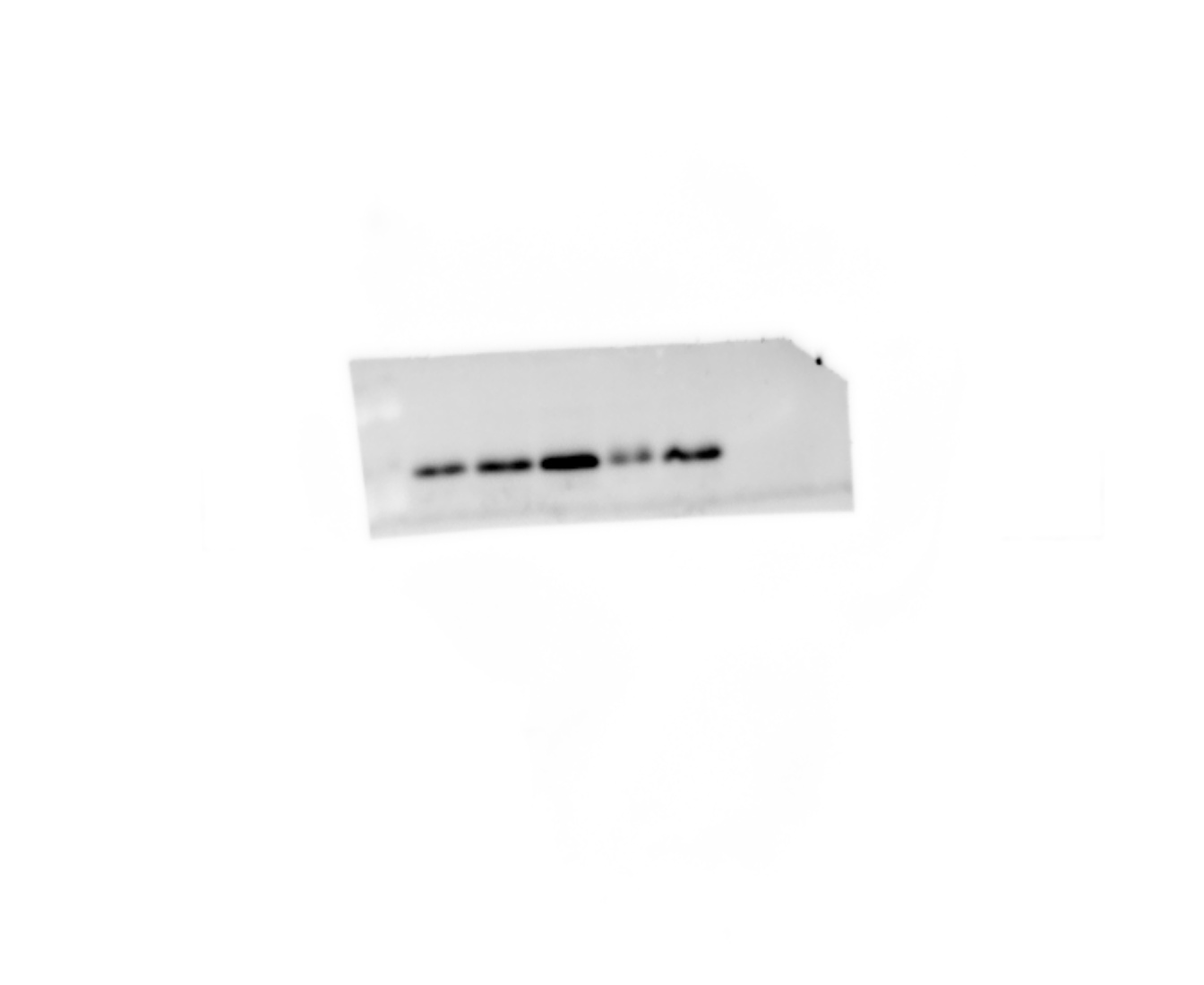

Supplement: Supplementary file 1 — Additional file 1. [file 12885_2022_9459_MOESM1_ESM.zip › Supplementary Information/WB/Figure 8C PC9 bax.jpg]

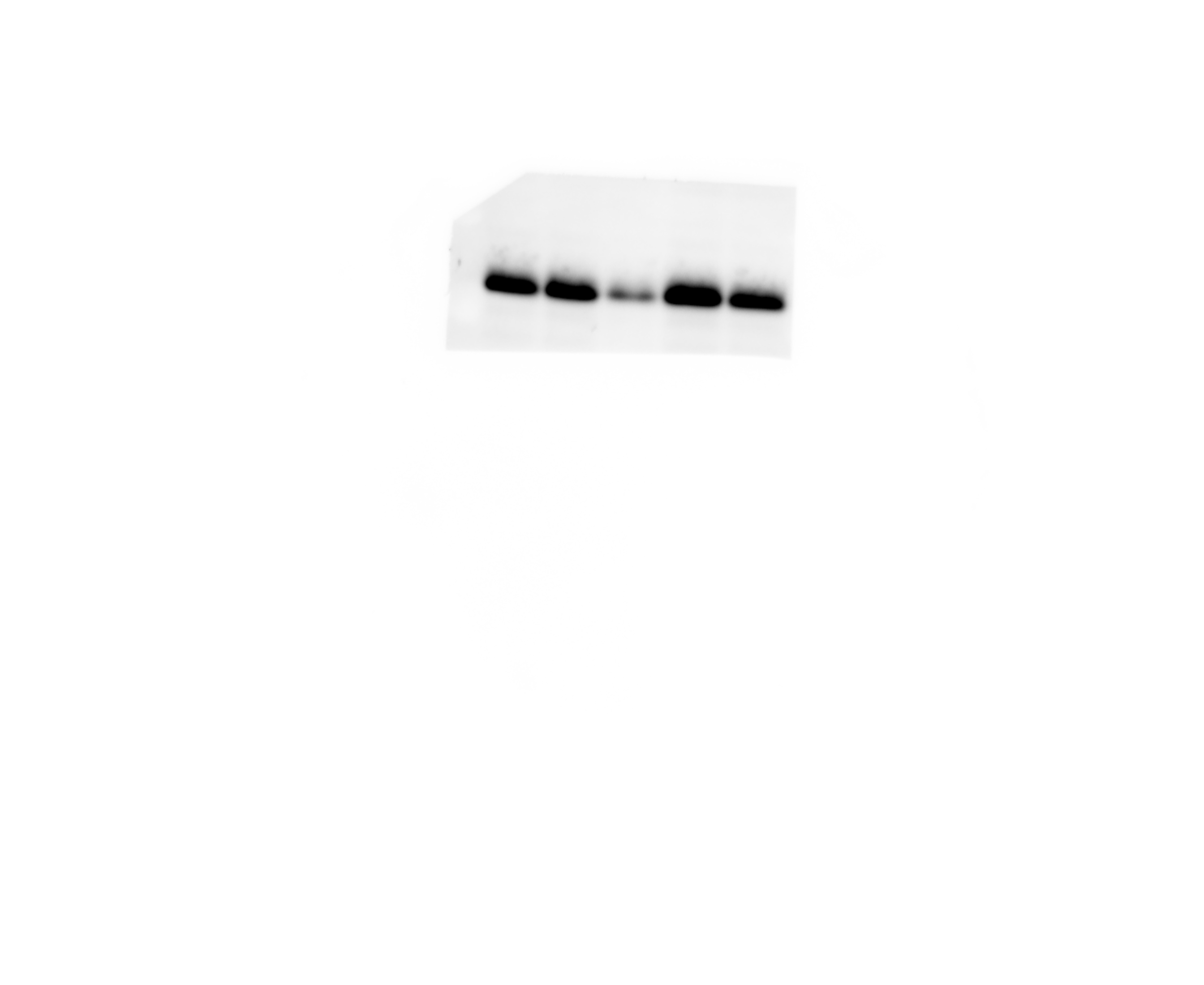

Supplement: Supplementary file 1 — Additional file 1. [file 12885_2022_9459_MOESM1_ESM.zip › Supplementary Information/WB/Figure 8C PC9 bcl-2.jpg]
